# MicroRNAs and the Diagnosis of Childhood Acute Lymphoblastic Leukemia: Systematic Review, Meta-Analysis and Re-Analysis with Novel Small RNA-Seq Tools

**DOI:** 10.3390/cancers14163976

**Published:** 2022-08-17

**Authors:** Ioannis Kyriakidis, Konstantinos Kyriakidis, Aspasia Tsezou

**Affiliations:** 1 Laboratory of Cytogenetics and Molecular Genetics, Faculty of Medicine, School of Health Sciences, University of Thessaly, 41500 Larissa, Greece; 2 Laboratory of Biological Chemistry, School of Medicine, Aristotle University of Thessaloniki, 54124 Thessaloniki, Greece; 3 Genomics and Epigenomics Translational Research (GENeTres), Center for Interdisciplinary Research and Innovation, Aristotle University of Thessaloniki, 57001 Thessaloniki, Greece; 4 Department of Biology, School of Health Sciences, University of Thessaly, 41500 Larissa, Greece

**Keywords:** microRNA, non-coding RNA, acute lymphoblastic leukemia, child, biomarker, diagnosis, subtype classification, disease susceptibility, sequence analysis

## Abstract

**Simple Summary:**

MicroRNAs (miRNAs) have been under the spotlight for the last three decades. These non-coding RNAs seem to be dynamic regulators of mRNA stability and translation, in addition to interfering with transcription. Circulating miRNAs play a critical role in cell-to-cell interplay; therefore, they can serve as disease biomarkers. Meta-analysis of published data revealed that the CC genotype of rs4938723 in pri-miR-34b/c and the TT genotype of rs543412 in miR-100 confer protection against acute lymphoblastic leukemia (ALL) in children. Reanalysis of small RNA-seq data with novel tools identified significantly overexpressed members of the miR-128, miR-181, miR-130 and miR-17 families and significantly lower expression of miR-30, miR-24-2 and miR143~145 clusters, miR-574 and miR-618 in pediatric T-ALL cases compared with controls. Inconsistencies in methodology and study designs in most published material preclude reproducibility, and further cohort studies need to be conducted in order to empower novel tools, such as ALLSorts and RNAseqCNV.

**Abstract:**

MicroRNAs (miRNAs) have been implicated in childhood acute lymphoblastic leukemia (ALL) pathogenesis. We performed a systematic review and meta-analysis of miRNA single-nucleotide polymorphisms (SNPs) in childhood ALL compared with healthy children, which revealed (i) that the CC genotype of rs4938723 in pri-miR-34b/c and the TT genotype of rs543412 in miR-100 confer protection against ALL occurrence in children; (ii) no significant association between rs2910164 genotypes in miR-146a and childhood ALL; and (iii) SNPs in DROSHA, miR-449b, miR-938, miR-3117 and miR-3689d-2 genes seem to be associated with susceptibility to B-ALL in childhood. A review of published literature on differential expression of miRNAs in children with ALL compared with controls revealed a significant upregulation of the miR-128 family, miR-130b, miR-155, miR-181 family, miR-210, miR-222, miR-363 and miR-708, along with significant downregulation of miR-143 and miR-148a, seem to have a definite role in childhood ALL development. MicroRNA signatures among childhood ALL subtypes, along with differential miRNA expression patterns between B-ALL and T-ALL cases, were scrutinized. With respect to T-ALL pediatric cases, we reanalyzed RNA-seq datasets with a robust and sensitive pipeline and confirmed the significant differential expression of hsa-miR-16-5p, hsa-miR-19b-3p, hsa-miR-92a-2-5p, hsa-miR-128-3p (ranked first), hsa-miR-130b-3p and -5p, hsa-miR-181a-5p, -2-3p and -3p, hsa-miR-181b-5p and -3p, hsa-miR-145-5p and hsa-miR-574-3p, as described in the literature, along with novel identified miRNAs.

## 1. Introduction

**Figure 1:**
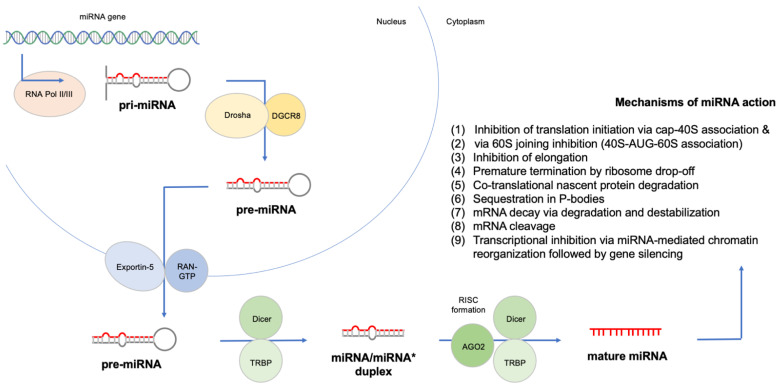
Canonical biogenesis and mechanism of action of miRNAs.

Acute lymphoblastic leukemia (ALL) remains the predominant cancer type of childhood; despite the advancements in ALL treatment and genetic characterization, there is a need for reliable biomarkers to further improve survival beyond 93% [1,2,3,4,5]. Conventional diagnostics of ALL are based on suggestive findings in peripheral blood confirmed by bone marrow aspiration and biopsy, flow cytometry, cytogenetics and molecular studies. Chemotherapy and radiation therapy are the cornerstones of childhood ALL treatment, whereas targeted therapies have been recently added to the clinicians’ armamentaria with favorable outcomes [5,6]. The discovery of the first microRNA (miRNA) in 1993 was succeeded by numerous studies on the role of miRNAs in disease. MicroRNAs are regulatory small non-coding RNAs, and their mature form is an average of 22 nucleotides in length. According to the latest miRBase release (http://mirbase.org/, accessed on 16 August 2022), 1917 human miRNAs have been documented to date, most of which have distinct biological features and gene targets. miRNAs were recently identified as key regulators of lymphoid differentiation, playing a major role in leukemia biology. Upregulated miRNAs and downregulated miRNAs seem to act as oncogenes and tumor suppressor genes, respectively, whereas miRNA signatures can be utilized to recognize ALL patients and discrimination ALL subtypes. Figure 1 displays the canonical pathway of miRNA biogenesis and describes their mechanism of action [7,8,9]. We conducted a review of the literature, in addition to a meta-analysis of miRNA single-nucleotide polymorphisms (SNPs) and a reanalysis of small RNA-seq datasets, to investigate and highlight the potential role of miRNAs as biomarkers of childhood ALL.

## 2. Materials and Methods

We have searched PubMed, Scopus and Cochrane Library for “[(microRNA or miRNA) AND (childhood) AND (lymphoblastic leukemia)]” until March 2022 using the corresponding MeSH terms. Abstracts from both International Society of Paediatric Oncology (SIOP) and American Society of Hematology (ASH) meetings were also scanned for relevant content. An additional search was conducted for every miR of interest through HMDD v3.2: the Human microRNA Disease Database (http://www.cuilab.cn/hmdd, accessed on 16 August 2022) and miRbase. References of extracted studies were thoroughly scanned for relevant literature.

We further reanalyzed data from the only available small RNA-seq study that included bone marrow (BM) samples from 34 children with T-ALL at diagnosis, along with 5 BM samples from healthy donors [10,11]. Samples were processed using the small RNA-seq pipeline implemented in the bcbio-nextgen project [12]. Raw reads were examined for quality issues using FastQC (http://www.bioinformatics.babraham.ac.uk/projects/fastqc/, accessed on 16 August 2022) to ensure that library generation and sequencing were suitable for further analysis. Adapter sequences were detected using DNApi [13]. Adapter sequences and other contaminant sequences, such as polyA tails and low-quality sequences with PHRED quality scores <5, were trimmed from reads using Atropos [14]. Trimmed reads were aligned to Homo sapiens GRCh38 miRBase v21 with SeqBuster [8,15]. The aligned reads were used with SeqCluster to characterize the whole small RNA transcriptome and classify reads into rRNA, miRNA, repeats, genes, tRNAs and others with UCSC annotation [16,17]. Alignments were checked for evenness of coverage, rRNA content, genomic context of alignments (for example, alignments in known transcripts and introns), complexity and other quality checks using a combination of FastQC and MultiQC custom codes inside the bcbio-nextgen pipeline [18]. Data were loaded into R using the bcbioSmallRna R package and isomiRs’ Bioconductor package to obtain normalized expression values. Small RNA-seq differential expression analysis was performed using DESeq2 [19].

Review Manager (RevMan) version 5.4.1 (The Cochrane Collaboration, London, UK) was used to validate and investigate miRNA gene SNPs with respect to susceptibility to childhood ALL that had published results in more than one study.

## 3. Results

A total of 73 studies were identified based on the inclusion criteria (only pediatric population, only human samples and only case–control studies involving miRNAs): 62 publications concerned differential expression of miRNAs (19 with only one type of ALL and 43 regarding ALL cases of all types; Table 1), whereas 14 studies were focused on predisposition to ALL based on SNPs in known miRNAs (Table 2). Four studies had mixed adult and pediatric population samples, but their results will only be discussed and not be further analyzed [20,21,22,23]. The heterogeneity between the above studies is remarkable, and previous reviews of the field have failed to be accurate [24].

### 3.1. miRNA Polymorphisms and Childhood ALL

As mentioned above, polymorphisms in several miRNA genes have been implicated in childhood ALL pathogenesis (Table 2). The aim of this systematic review is to investigate the role of microRNA SNPs in childhood ALL occurrence, adhering to the Preferred Reporting Items for Systematic Reviews and Meta-Analyses guidelines (PROSPERO registration: 353499). With respect to SNPs, contradictory results have been published for miR-146a, miR-196a-2, miR-499 and miR-612 genes. A previous meta-analysis reported increased risk for childhood ALL in CC carriers of miR-146a rs2910164 in Asian populations, although significant association has only been found when using the additive model (CC vs. GG: OR, 1.598; 95% CI: 1.003 to 2.545; *p* = 0.049) and no other model [87]. We investigated all six studies included in the latter systematic review (one study reported increased risk for childhood ALL in carriers of the CC genotype of miR-146a rs2910164, one study found increased risk for CC and CG genotypes and one study supported the protective role of GG genotype, whereas four studies revealed non-significant associations), plus one study from Brazil that found no significant association of miR-146a rs2910164 genotypes with susceptibility to childhood ALL [88].

**Figure 2:**
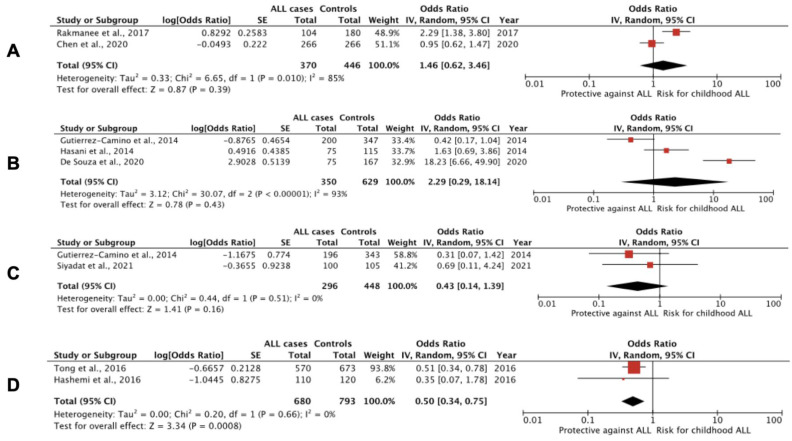
Forest plots of studies, including results for SNPs in miRNA genes in childhood ALL cases and controls. (**A**) T>C polymorphism of rs11614913 in the miR-196a-2 gene: CC vs. CT+CC; (**B**) A>G polymorphism of rs37464444 in miR-499 gene -only B-ALL cases in de Souza et al, 2021, and Gutierrez-Camino et al., 2014 [88,91]: GG vs. GA+AA; (**C**) G>A polymorphism of rs12803915 in the miR-612 gene: AA vs. AG+GG; (**D**) T>C polymorphism of rs4938723 in the pri-miR-34b/c gene: CC vs. CT+TT.

We performed a meta-analysis including the seven studies mentioned above regarding data for rs2910164 genotypes in miR-146a and found no significant associations for any of the models. The additive model that had been previously utilized concluded that children carrying the CC genotype of miR-146a rs2910164 had a 50% increased chance of being diagnosed with ALL compared with GG carriers (OR, 1.52; 95% CI: 0.93 to 2.49; I^2^ = 75%), but this association was not significant (*p* = 0.09). SNP rs57095329 A>G in miR146A has been previously associated with reduced binding of the transcription factor V-Ets oncogene homolog 1 (Ets-1) and decreased miR-146a expression, whereas rs2910164 G>C seems to reduce pre-miR-146a nuclear-processing efficiency, thus reducing the expression of mature miR-146a and resulting in less efficient inhibition of the target genes (by affecting binding specificity), including tumor necrosis factor (TNF), TNF receptor-associated factor 6 (TRAF6) and interleukin 1 receptor-associated kinase 1 (IRAK1) [47,89]. On the other hand, miR-146a overexpression (i.e., with G allele) can inhibit *IRAK1* and *TRAF6* expression, impair the activity of their downstream molecule, NF-ĸB, and suppress several NF-ĸB-targeted inflammatory genes, such as *IL6*, *CXCL8* alias *IL-8*, *IL1B* and *TNF* [90]. With respect to other repeatedly studied miRNA SNPs and predisposition to childhood ALL, only the CC genotype of rs4938723 in pri-miR-34b/c has been linked significantly with protection against the disease. Forest plots of studies including results for SNPs in miRNA genes in childhood ALL cases and controls are shown in Figure 2. In summary, our systematic review and meta-analysis showed that SNPs in pri-miR-34b/c and miR-100 genes have been linked with protection again childhood ALL, whereas SNPs rs3805500 in *DROSHA*, rs3746444 in miR-449b, rs2505901 in miR-938, rs12402181in miR-3117 and rs62571442 in miR-3689d-2 genes have been associated with susceptibility to B-ALL (Table 2).

**Table 2:** Predisposition to childhood ALL and miRNA SNPs.

Study	Cohort Characteristics	Methods	Outcomes
Siyadat et al., 2021 [92]	100 childhood B-ALL patients and 105 controls; Iran	RT-PCR, HRM and Sanger sequencing; BM or whole blood	No significant relationship was confirmed between rs12803915 variants of miR-612 and susceptibility to B-ALL.
Pei et al., 2020 [90]	266 childhood ALL patients and 266 controls; Taiwan	PCR-RFLP; whole blood	The G allele of **miR-146a** polymorphism rs2910164 seems to be a protective biomarker for childhood ALL (OR, 0.66; 95% CI: 0.52 to 0.85; *p* = 0.0011), whereas patients with the GG genotype were associated with decreased susceptibility to ALL (OR, 0.4; 95% CI: 0.23 to 0.67; *p* = 0.0004) compared with healthy controls. Conspicuously, children bearing CG and GG genotypes were found to have earlier onset compared to those with CC genotype (*p* = 0.025 and *p* = 0.0001, respectively). The G>C polymorphism leads to reduced amounts of mature miR-146a, whereas the C allele has reduced capacity to inhibit target genes.
C.C. Chen et al., 2020 [93]	266 childhood ALL patients and 266 controls; Taiwan	PCR-RFLP; whole blood	The T>C polymorphism of rs11614913 in miR-196a-2 does not seem to be associated with susceptibility to childhood ALL.
De Souza et al., 2020 [88]	100 childhood B-ALL patients and 180 adult controls; Brazil	qRT-PCR; whole blood	The mutant homozygote (AA) genotype of ***DROSHA*** rs3805500 was associated with a threefold increase in the risk of developing ALL (recessive model: GG + GA vs. AA; OR, 2.913; 95% CI: 1.415 to 5.998; *p* = 0.004). The mutant homozygote (GG) genotype of the **miR-499** gene rs3746444 was associated with a 17.8-fold increase in the risk of developing ALL (recessive model: AA + AG vs. GG; 95% CI: 5.55 to 57.016; *p* < 0.001). In contrast, the wild homozygous (CC) genotype of the **miR-938** gene rs2505901 seems to confer a protective effect against developing ALL (dominant model: CC vs. CT + TT; OR, 0.359; 95% CI: 0.160 to 0.805; *p* = 0.013). No significant associations for *AGO1* rs636832, *MIR219A1* rs213210 and rs107822, *MIR146A* rs2910164, *MIR330* rs12894467 and *MIR608* rs4919510.
Jemimah Devanandan et al., 2019 [47]	71 childhood ALL patients and 74 controls; India	qRT-PCR; whole blood	No statistically significant association was found between miR-146a gene SNPs rs2910164 G>C and rs57095329 A>G and ALL risk.
Xue et al., 2019 [42]	831 childhood ALL patients and 1079 controls; validation: 88 childhood ALL cases and 99 controls; China	qRT-PCR; whole blood; plasma from validation cohort	Subjects carrying the mutant homozygous TT genotype of **miR-100** rs543412 had a statistically significantly decreased risk of childhood ALL (OR, 0.73; 95% CI: 0.55 to 0.97; *p* = 0.029). No association was been found for miR-146a rs2910164 and miR-210 rs7395206 polymorphisms and ALL.
Gutierrez-Camino et al., 2018 [94]	217 children with B-ALL and 330 controls from Spain and 75 children with B-ALL and 96 controls from Slovenia	GoldenGate genotyping assay and OpenArray genotyping; whole blood or BM	The AA genotype of rs12402181 in **miR-3117**-3p was associated with B-ALL risk (OR, 1.44; 95% CI: 1.01 to 2.08; *p* = 0.047; GG vs. AG vs. AA) in the Spanish cohort. The same effect was observed in the Slovenian cohort (OR, 2.01; 95% CI: 1.02 to 3.95; *p* = 0.041). When both populations were analyzed together, they displayed a more significant trend (OR, 1.53; 95% CI: 1.12 to 2.09; *p* = 0.006). With respect to allele frequency analysis, minor allele A showed a 1.51-fold increased risk of B-ALL in total (95% CI: 1.11 to 2.05; *p* = 0.007). The CT/CC genotype in rs62571442 of **miR-3689d-2** was associated with a significantly increased risk of developing B-ALL (OR, 1.48; 95% CI: 1.02 to 2.15; *p* = 0.039) in the Spanish cohort, whereas the risk was even greater in the Slovenian cohort (OR, 3.57; 95% CI: 1.57 to 8.12; *p* = 0.001). Analyzed together, both cohorts confirmed the latter association (OR, 1.31; 95% CI: 1.06 to 1.60; *p* = 0.011). Allele frequency analysis in both populations showed that minor allele C is associated with a 1.31-fold increased risk of B-ALL (95% CI: 1.06 to 1.6; *p* = 0.012).
Liu et al., 2018 [95]	200 childhood ALL patients and 100 controls; China	RFLP; whole blood	The **miR-146a** rs2910164 CC or CG genotype significantly increased the risk of ALL (frequency of GG, GC and CC genotypes in the patient group and in the control group was 16%, 44.5%, 39.5%, and 29%, 41%, 30%, respectively). The expression of GC/CC genotypes were significantly higher in patients than in controls (GG genotype as reference; for the GC genotype: OR, 1.967; 95% CI: 1.054 to 3.672; *p* = 0.037; and for the CC genotype: OR, 2.386; 95%CI: 1.239 to 4.595; *p* = 0.012).
Rakmanee et al., 2017 [96]	104 childhood ALL patients and 180 controls; Thailand	PCR-RFLP; whole blood	Variant CC of rs11614913 in **miR-196a-2** (OR, 4.321; 95% CI: 2.091 to 8.930; *p* < 0.001), TC heterozygote (OR, 2.248; 95% CI: 1.103 to 4.579; *p* = 0.024) and CC+TC genotypes (OR, 2.921; 95% CI: 1.504 to 5.673; *p* = 0.001) were associated with childhood ALL susceptibility compared with the TT wild type. CC homozygotes were associated with significantly increased miR-196a-2 expression.
Chansing et al., 2016 [97]	100 childhood ALL patients and 200 controls; Thailand	PCR-RFLP; whole blood	There was no association between miR-146a rs2910164 G>C polymorphism and susceptibility to childhood ALL.
Hashemi et al., 2016 [98]	110 childhood ALL patients and 120 controls; Iran	PCR-RFLP; whole blood	A study of polymorphism rs4938723 in **pri-miR-34b/c** in ALL and healthy children revealed decreased risk of ALL in heterozygous (OR, 0.48; 95% CI: 0.28 to 0.84; *p* = 0.012; TC vs. TT), and overdominant (OR, 0.51; 95% CI: 0.3 to 0.89; *p* = 0.02; TC vs. TT+CC) inheritance models. The C allele significantly decreased the risk of childhood ALL compared to the T allele (OR, 0.52; 95% CI: 0.33 to 0.83; *p* = 0.006).
Tong et al., 2016 [99]	570 childhood ALL patients at diagnosis and 673 controls; China	RT-PCR; whole blood	The CC genotype in rs4938723 of **pri-miR-34b/c** was associated with significantly reduced ALL risk (CC vs. TT: OR, 0.51; 95% CI: 0.33 to 0.8; *p* = 0.003, and CC vs. TT+TC: OR, 0.49; 95% CI: 0.32 to 0.75; *p* = 0.002).
Gutierrez-Camino et al., 2014 [91]	213 childhood precursor B-ALL patients and 387 adult controls; Spain	OpenArray; remission BM or whole blood	The A allele of rs12803915 in premature **mir-612** was found to be protective (GG vs. GA vs. AA; OR, 0.61; 95% CI: 0.42 to 0.88; *p* = 0.007) against ALL risk. The G allele of rs3746444 in the seed region of mature **miR-499**-3p was also found to be protective (AA vs. AG vs. GG; OR, 0.67; 95% CI: 0.49 to 0.91; *p* = 0.009). An association with rs10061133 in **mir-449b** was also identified (AA vs. GA+GG; OR, 0.52; 95% CI: 0.31 to 0.89; *p* = 0.012). Eight SNPs present in the six miRNA biogenesis pathway genes (*TNRC6B*, *DROSHA*, *DGCR8*, *EIF2C1* or *AGO1*, *CNOT1* and *CNOT6*) were also identified.
Hasani et al., 2014 [89]	75 childhood ALL patients and 115 controls; Iran	T-ARMS-PCR; whole blood	The G>C variant of rs2910164 in hsa-**miR-146a** was found to significantly increase risk of ALL (CC vs. GG; OR, 4.24; 95% CI: 1.52 to 11.87; *p* = 0.006, and GC vs. GG; OR, 3.55; 95% CI: 1.41 to 8.93; *p* = 0.007; C vs. G; OR 1.73; 95% CI: 1.13 to 2.67; *p* = 0.012). No association was found between rs3746444 of miR-499 and ALL risk.

AGO1 = argonaut RISC component 1; CNOT1/6 = carbon catabolite repression 4 protein-NOT transcription complex subunit 1/6; DGCR8 = DiGeorge syndrome critical region gene 8 Microprocessor complex subunit; HRM = high-resolution melt analysis; RFLP = restriction fragment length polymorphism; T-ARMS = tetra-primer amplification refractory mutation system; TNRC6B = trinucleotide repeat-containing adaptor 6B. MicroRNAs in bold indicate statistically significant results.

**Figure 3:**
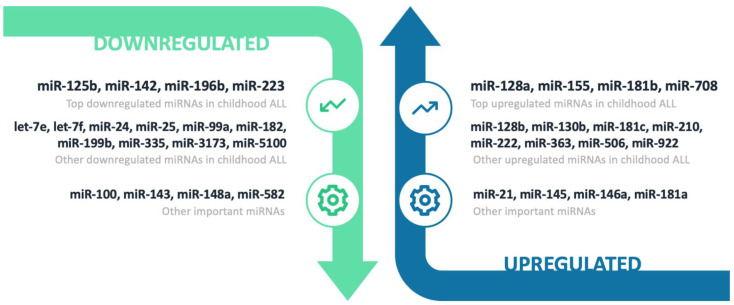
Schematic representation of significantly differentially expressed miRNAs in childhood ALL diagnosis compared to controls.

Analysis of all 43 publications with data for miRNA expression in pediatric ALL patients versus controls revealed significant differential expression for several miRNAs (Figure 3). The top list was built according to published data and emphasis was placed on studies with a large sample size (*n* > 100), studies with validated results (e.g., microarrays validated by qRT-PCR in the same or another cohort), studies that used next-generation sequencing methods (e.g., RNA-seq) and studies with minimal risk of bias (e.g., exclusion of studies with adult or sick controls, studies with ALL cases undergoing chemotherapy, studies with disproportionate numbers of B- and T-ALL cases, etc.).

### 3.2. Downregulated miRNAs

#### 3.2.1. miR-125b

Downregulation of miR-125b was reported in four case–control studies (two of them with more than 100 cases), whereas one study reported otherwise [51,57,77,83]. Some pediatric BCP-ALL cases displayed upregulation of miR-125b: *ETV6*-*RUNX1*, *BCR*-*ABL1*, *ERG*-deregulated and t(11;14)(q24;q32) cases [29,46,100,101,102,103,104]. Although pediatric T-ALL cases are associated with lower miR-125b levels, *TLX1*- and *TLX3*-driven cases seem to have higher levels compared to other T-ALL cases, and infants with T-ALL have significantly higher miR-125b levels than the respective childhood cases [57,105,106]. miR-125b belongs to a family comprising eight members: miR-10a, miR-10b, miR-99a, miR-99b, miR-100, miR-125a, miR-125b-1 and miR-125b-2. In humans, miR-125 homologs are organized into three distinct clusters: (i) miR-125b-1, let-7a-2 and miR-100 in chromosome 11; (ii) miR-125b-2, let-7c and miR-99a in chromosome 21; and (iii) miR-125a, let-7e and miR-99b in chromosome 19; this suggests that let-7 and miR125 families have a cooperative biological function related to leukemogenesis. The diverse role of miR-125 in hematopoiesis relies on the fact that it enhances proliferation of early progenitors but also blocks their further differentiation [103]. A significant elevation of miR-125b levels after induction of treatment compared with diagnosis levels is noted, but elevated day 33/diagnosis miR-125b ratio has been linked with relapse and poor outcome [57]. Extra genetic material with respect to chromosome 21 (high hyperdiploid, Down syndrome and iAMP ALL) does not seem to contribute to increased expression levels of the miR-125b cluster, whereas its upregulation is typically featured in *ETV6*-*RUNX1* cases, conferring survival advantage to growth inhibitory signals, independent of p53. Moreover, knockdown of miR-125b in cell lines bearing the previous fusion demonstrated increased apoptosis rates after treatment with either doxorubicin or staurosporine [101]. With respect to *TLX3*-positive cases, overexpression of miR-125b and miR-99a provokes the transactivation of the lncRNA gene *LINC00478*, which hosts the miR-125b-2 cluster and therefore leads to differentiation arrest and expansion of transformed T-cells [106]. In addition, a recent study showed that circPVT2 can promote ALL cell proliferation, migration and invasion by reducing miR-125b and enhancing the NF-κB signaling pathway [107]. Upregulation of miR-125b in childhood ALL is associated with the methylation of the promoter regions of tumor suppressor genes *PPP1CA* (encoding protein phosphatase 1 catalytic subunit alpha), *BTG2* (gene of B-Cell translocation gene anti-proliferation factor 2) and *PTEN* (encoding phosphatase and tensin homolog) and their downregulation (reversed by DNA methyltransferase inhibitor decitabine) [108]. In vitro experiments support the oncogenic role of miR-125b, as it seems to repress *BCL2* (and thus promote proliferation in response to CD40 ligand, i.e., CD154 in human leukemic B-cells) and to enhance proliferation and block differentiation by targeting *ARID3A* (AT-rich interaction domain 3A or BRIGHT, i.e., B-cell regulator of immunoglobulin heavy-chain transcription) in t(11;14)(q24;q32) B-ALL [109,110].

#### 3.2.2. miR-142

Significant downregulation of miR-142 in children with ALL compared with controls was reported in three studies [40,54,86], whereas two other study groups reported a significant upregulation of this miRNA [77,83]. Significant upregulation of miR-142 has also been reported in T-ALL, pre-B-ALL, *KMT2A*-r and B-other cases compared to controls [79,83,85]. miR-142 clusters with miR-4736 in chromosome 17 and targets *BCLAF1* (which encodes BCL2-associated transcription factor 1) and *KMT2A*-*AFF1* fusion protein (formerly known as *MLL*-*AF4*) in t(4;11) leukemia [77,111]. Expression of miR-142 has been associated with glucocorticoid (GC) resistance in ALL, mainly by direct targeting of 3′-UTR of GRα mRNA (glucocorticoid receptor alpha encoded by *NR3C1*) [112,113]. The oncogenic role of miR-142 in T-ALL (relatively resistant to GCs) is supported by the findings that high miR-142 expression results in low levels of cAMP (cyclic adenosine monophosphate) and weak protein kinase A (PKA) activity, thus alleviating its inhibitory effect on T-cell proliferation [113]. An early report of an aggressive B-ALL case bearing a masked t(8;17) translocation indicated overactive *MYC* as a result of miR-142 promoter rearrangement [114]. Moreover, a murine model demonstrated that miR-142 knockdown results in altered lymphopoiesis and immunodeficiency [115].

#### 3.2.3. miR-196b

Downregulation of miR-196b is a common finding in childhood ALL [35,54,69,76,78,82,83]. Conversely, upregulation of miR-196b is prominent in pediatric *KMT2A*-r cases [29,83,100,116] and in some T-ALL subgroups (such as *HOXA*-r, *PICALM*-*MLLT10* or *CALM*-*AF10*, inv(7)(p15q35) and *SET*-*NUP214* cases), especially when aberrant expression of *HOXA* genes is evident [20,83,116]. *HOXA9* and *MEIS1* homeobox oncogenes (adjacent and coexpressed with miR-196b), along with *FAS* tumor suppressor, are direct targets of miR-196b and drive carcinogenesis in *KMT2A*-r cases in a bidirectional manner [117]. As expected, miR-196b levels seem to be significantly higher in pediatric T-ALL compared with B-ALL cases, whereas miR-196b is significantly overexpressed in children with T-ALL compared with infantile T-ALL cases [76,105,116,118]. The tumor-suppressive role of miR-196b is supported by the findings that it regulates *MYC* and *ERG* and that it binds the 3′-UTR region of *IGFBP3* mRNA (reversible by resveratrol in vitro) [78,119]. A ChIP (chromatin immunoprecipitation) study of 179 children with ALL from Spain reported high levels of K9H3me2 and/or low levels of K4H3me3 near to CpG islands in the miR-196b gene, indicating its downregulation. Treatment with decitabine led to the successful upregulation of its levels in vitro, suggesting a major role of methylation status in early leukemogenesis [120].

#### 3.2.4. miR-223

Downregulation of miR-223 in childhood ALL compared with controls has been disclosed multiple times in the literature [34,46,54,69,77,84]. Only one small study has reported otherwise [40]. An inconsistent finding is that many childhood T-ALL subgroups exhibit a different profile with considerable overexpression of miR-223 compared with controls: *TLX1*- and *TLX3*-deregulated, *SIL*-*TAL1*, *PICALM*-*MLLT10* and inv(7) cases [79]. Nevertheless, two studies involving children with T-ALL reported downregulation of miR-223 compared to healthy subjects [10,20]. In the same context, infants with T-ALL have been found to have significantly higher miR-223 levels than childhood T-ALL cases, whereas high levels of miR-223 in *KMT2A*-r cases are apparent [79,105]. Hyperdiploid cases display the highest and *TCF3*-r cases (E2A or Transcription Factor 3), the lowest miR-223 levels among BCP-ALL cases [29,100]. Notwithstanding, low expression of plasma miR-223 at admission has been associated with poor prognosis in childhood ALL [121]. This miRNA seems to inhibit cell proliferation, migration and invasion, and to promote apoptosis by targeting *FOXO1* (encoding forkhead box O1 protein) [34]. Expression of miR-223 is uniformly downregulated in B-cell lymphoproliferative disorders and is stage-specific in B-cell differentiation, with higher levels in naive and memory cells compared with germinal center cells [122]. Furthermore, an early in vitro study demonstrated that miR-223 can reversibly regulate erythroid and megakaryocytic differentiation via downmodulation of *LMO2* [123]. Conversely, a study involving pediatric samples reported that *TAL1*-mediated upregulation of miR-223 leads to promotion of a malignant phenotype in T-ALL through repression of the *FBXW7* tumor suppressor (encoding F-box and WD repeat domain-containing 7 protein) [124]. In particular, elevated MYB levels in T-ALL can arise either directly through TCR-mediated *MYB* proto-oncogene translocations, *MYB* duplications and enhanced TAL1 complex binding at the *MYB* locus or indirectly through the TAL1/miR-223/FBXW7 regulatory axis [125]. A study including children suggested myeloid-specific features for miR-223, reporting significantly lower expression in ALL compared with AML samples [126]. Intriguingly, monitoring of miR-223 demonstrated significantly higher levels in 13 children (3 with T-ALL and 10 with BCP-ALL) after 24 h of GC monotherapy compared with their diagnosis levels [127].

#### 3.2.5. Other Downregulated miRNAs

Significantly lower expression of 2 of the 12 members of the let-7 gene family, i.e., let-7e and let-7f-1, has been implicated in childhood ALL pathogenesis. MicroRNA let-7e is clustered with miR-99b and miR-125a, let-7f-1 is clustered with let-7a-1 and let-7d and let-7f-2 is clustered with miR-98 [59,76,83]. The let-7 family is known for its tumor-suppressor activity (by inhibiting *NRAS*, *HMG2A* and *MYC* protooncogenes), and their reduced levels are typically associated with cancer stemness [128,129]. In the same context, significantly lower levels of let-7b and let-7d compared with controls have been documented in children with *KMT2A*-r ALL and B-ALL, respectively [67,69,130]. Conversely, let-7c, originating from chromosome 21 and clustered with miR-99a, seems to be upregulated in pediatric ALL cases involving fusions in this chromosome, i.e., *ETV6*-*RUNX1*- and *ERG*-related cases [100,101,102]. Whereas miR-99a is generally downregulated in pediatric ALL, its upregulation in BCP-ALL cases has been identified as the strongest indicator of *ETV6*-*RUNX1* fusion presence [29,100,101]. Restoration of underexpressed levels of miR-99a and miR-100 in ALL cells in vitro resulted in suppression of cell proliferation and increased cell apoptosis after dexamethasone treatment. Moreover, elevated expression of miR-99a and miR-100 seems to target the FKBP5 (FK506-binding protein prolyl isomerase 5) signaling pathway (in turn influence GC receptor activity) and to inhibit expression of *IGF1R* (insulin-like growth factor 1 receptor) and *MTOR* (mechanistic target of rapamycin kinase), along with their downstream *MCL1* (myeloid cell leukemia sequence 1 apoptosis regulator, BCL2 family member) oncogene [75].

Downregulation of miR-24 has also been suggested as a hallmark of ALL pathogenesis in children [70,86]. Two studies on children with T-ALL reported significantly reduced expression of miR-24 compared with healthy children [10,68]. On the contrary, significantly elevated miR-24 levels in children with ALL compared with controls were reported in recent small study in Egypt [30]. The miR-24 miRNA family consists of miR-24-1 (clustered with miR-23b, miR-27b and miR-3074 in chromosome 9) and miR-24-2 (clustered with miR-23a and miR-27a in chromosome 19). Low miR-24 expression was previously associated with a diminished tumor-suppressor effect of miR-31 and *PAX5* (paired box 5 protein) deletion. In the same study, high expression of miR-24 was associated with *PAR1* (*F2R*; encoding coagulation factor II thrombin receptor) deletion [131]. Beyond BCL2, miR-24-2 was demonstrated to target a number of proteins functionally important for cell survival, such as YWHAZ (tyrosine 3-monooxygenase/tryptophan 5-mono-oxygenase activation protein zeta), TP53 (Tumor Protein P53), SMAD3 (SMAD Family Member 3), ESR1 (Estrogen Receptor 1) and CREBBP (binding protein of cAMP-response element binding protein) [132]. As demonstrated by acute leukemia cell lines and primary samples (of unknown age and leukemia type), the expression of the miR-24-2 cluster was found to be significantly decreased compared to CD34^+^ PBMCs from normal adult donors (86% of pre-B-ALL and 100% of T-ALL) [133]. A study on acute erythroid leukemia suggested that the miR-24-2 cluster mediates the inactivation of multiple targets in the IL6ST/JAK1/STAT3 pathway (interleukin 6 cytokine family signal transducer or GP130, Janus kinase 1 and signal transducer and activator of transcription 3, respectively) and promotes *GATA1* expression, thereby facilitating apoptosis and restraining adverse proliferation. The same study pointed out that JAK1 inhibitor ruxolitinib can rescue phenotypic changes induced by low miR-24-2 [134]. Significant downregulation of miR-25 has been also reported in pediatric ALL cases compared with healthy controls [59,62]. miR-24-2 belongs to a family comprising miR-92a-1, miR-92a-2 and miR-92b, whereas it is found clustered with miR-93 and miR-106b. On the contrary, a recent study identified miR-25 as a suitable endogenous normalizer for qRT-PCR in T-ALL [135].

As discussed below, miR-182 was found to be upregulated in some T-ALL subgroups (Supplementary Table S1), although its downregulation seems to efficiently discriminate pediatric ALL cases from healthy controls [50]. miR-182 clustered with miR-96, and miR-183 seems to be involved in GC treatment response by targeting FOXO3A (forkhead box O3 protein) and its downstream target, BIM (alias BCL2L11) [112,136]. Significant overexpression of miR-182 (before treatment) was calculated in pediatric GC-sensitive ALL cases compared with GC-resistant cases [137]. In the same context, the BM miR-143/miR-182 ratio seems to be significantly decreased in childhood ALL patients at diagnosis, whereas it is increased in more than 90% of patients at the end of induction (EoI) [50]. On the contrary, GC resistance in T-ALL might be explained by the upregulation of glycolysis, oxidative phosphorylation, cholesterol synthesis, glutamic acid synthesis, fast growth rate and activation of PI3K/AKT/mTOR (phosphoinositide 3-kinases, AKT serine/threonine kinases and mTOR pathway) and the MYC-signaling pathways. Therefore, miR-182, along with miR-185, is likely to enhance GC sensitivity and GC receptor auto-upregulation by suppressing mTOR activity [137]. Altogether, restoration of miR-182 levels could be a promising therapeutic strategy for childhood ALL.

Significant downregulation of miR-199b in children with ALL compared with healthy children has been also noted by several studies [27,84,86]. This miRNA is clustered with miR-3154, which was also found to be significantly downregulated in children with T-ALL compared with controls [10]. Another significantly downregulated miRNA in childhood ALL is miR-335 [37,43,69]. Fluctuations of miR-335 levels among childhood ALL cytogenetic groups was also reported (highest in *ETV6*-*RUNX1*- and lowest in *BCR*-*ABL1*-positive cases) [138]. It has been also reported that miR-335 specifically targets 3′-UTR of *RB1* (retinoblastoma transcriptional corepressor 1) and activates the p53 tumor-suppressor pathway [139]. Moreover, downregulation of miR-335 seems to decrease the sensitivity of ALL cells to prednisone (by enhancing its target *MAPK1*), whereas treatment with Ras-Raf-MEK-ERK pathway inhibitors can activate BIM and promote prednisolone-induced cell death [112,138].

Significant downregulation of miR-3173 and miR-5100 in children with ALL compared to healthy individuals has been documented in the literature, but data are scarce [59,61]. With respect to miR-3173, it has been shown to target the 3′-UTR of *PTK2* in B-ALL cells, whereas its downregulation seems to promote cell proliferation, migration and invasion via *PTK2* overexpression [61]. However, there are insufficient data to justify annotation confidence for miR-5100 [8].

As mentioned above, downregulation of miR-100 seems to be a hallmark of ALL development in children [38,75,76,83]. Paradoxically, one study reported significant upregulation of miR-100 in children with ALL compared with controls, although the study sample included a large proportion (33%) of biphenotypic ALL cases [65]. Among pediatric ALL cases, the highest levels of miR-100 have been measured in *ETV6*-*RUNX1*-positive cases, whereas miR-100 abundance is unlikely in hyperdiploid cases [29,76,100]. In addition, high miR-100 expression has been linked with low WBC levels (white blood cell levels; <50,000/mm^3^) at diagnosis and in vincristine- and daunorubicin-resistant cases (synergistically with miR-125b and miR-99a overexpression) [29,65,76,140].

Reduced expression of miR-143 has been associated with childhood ALL phenotype multiple times in the literature [10,28,50,84,86]. Most importantly, in vitro studies have illustrated a pathogenetic link between the notoriously poor prognosis of t(4;11) ALL and miR-143 elimination. Repression of miR-143 via methylation seems to enhance *KMT2A*-*AFF1* oncogene expression, whereas upregulation of this miRNA could be a therapeutic target for this ALL subgroup [141]. In the same context, miR-143 overexpression in ALL cells in vitro resulted in growth inhibition and induction of apoptosis by either silencing *DNMT3A* (DNA methyltransferase 3 alpha) or by downregulating *BCR*-*ABL1* expression in the respective cases [142,143]. A recent study reported significant upregulation of miR-145, which is clustered with miR-143, although this qRT-PCR study included only 13 pediatric ALL cases and 5 controls [36]. Regarding this cluster, genetic network analysis of 51 cancer types identified the miR-17~92 family as amplified and the miR-143~145 cluster as deleted, designating the latter cluster as a hallmark of tumorigenesis [144].

Significant reductions in miR-148a levels has been noted in children with ALL compared with controls [78,80,82,86]. A recent case–control study from Egypt supported the tumor-suppressive properties of miR-148a by calculating significant upregulation of miR-148a levels in treatment responders [30]. Many reports in recent years have established the role of miR-148a as a tumor suppressor through its ability to inhibit proliferation by directly suppressing *DNMT1* (encoding DNA methyltransferase 1) [145]. Copy-number loss in 7p15.2 of miR-148a was linked with adult ALL, whereas this miRNA downregulation seems to be characteristic of adult *KMT2A*-r cases [146]. The role of miR-582 downregulation in childhood ALL occurrence is debatable. The controversy lies on the fact that case–control studies have reported both significantly high and low expression of miR-582 in ALL cases compared to controls [10,20,83,86]. The dubious finding of significant upregulation of miR-582 in a study by Schotte et al. (2009) may be explained in part by the inhomogeneous sample (*KMT2A*-r cases: 22%) and the small number of controls (two children with brain tumors) [83].

### 3.3. Upregulated miRNAs

#### 3.3.1. miR-128 Family

This family consists of two members: miR-128-1 (formerly known as miR-128a), with its gene located in 2q21.3; and miR-128-2 (also known as miR-128b), with its gene residing in 3p22.3. Significant upregulation of both miR-128-1 and miR-128-2 has been associated with pediatric ALL cases compared with controls [10,28,46,53,68,76,83,84,86]. Two studies including both adults and children reported significant upregulation of miR-128 family members compared with normal CD19^+^ cells and endogenous snoRNA U6 content, respectively [126,147]. Among pediatric ALL cases, excessive upregulation of miR-128-1 is evident in t(4;11)- and *TCF3*-*PBX1*-positive cases, whereas the lowest values of miR-128-2 have been observed in *IKZF1* del, *PAX5* del and *KMT2A*-*AFF1* fusion-positive cases [76,131,148,149]. In agreement with the latter findings, significantly higher levels of both miRNAs were been documented in infantile T-ALL compared with childhood T-ALL cases [105]. Expression levels of miR-128-2 seem to play a pivotal role in GC resistance. Re-expression of miR-128-2 in ALL cell lines bearing the *KMT2A*-*AFF1* fusion resulted in sensitization to GCs in vitro, whereas target analysis has shown that miR-128-2 downregulates *KMT2A*, *AFF1* and their fusion gene [112,127,148,150]. A13G mutation in the *MIR128-2* gene seems to significantly reduce the processing of pri-miR-128-2 and therefore reduces dexamethasone-induced apoptosis, i.e., leads to GC resistance of t(4;11) ALL cells [150]. In addition, systemic GC monotherapy for 24 h resulted in significant downregulation of miR-128-2 levels in children with ALL, suggesting its critical role in GC response [127]. These oncomirs lead to leukemogenesis by affecting several pathways, mainly by inhibiting *TERT* (telomerase reverse transcriptase) mRNA, *PHF6* (PHD finger protein 6, especially in NOTCH1-driven T-ALL models), *BMI1* proto-oncogene and *UBE2W* (encoding ubiquitin-conjugating enzyme E2 W), an ubiquitin ligase involved in the BRCA DNA repair associated-pathway [46,149,151,152].

#### 3.3.2. miR-155

Upregulation of miR-155 is another important hallmark of childhood ALL, and many studies have cited significantly increased expression of miR-155 in pediatric ALL cases compared with healthy controls [25,27,28,36,39,44,46,62,68,69,77,86]. Among pediatric ALL cases, the lowest expression levels for miR-155 have been recorded in *ETV6*-*RUNX1*-positive and *ERG*-related cases (ETS transcription factor ERG or p55) [102,153]. This miRNA promotes the development of ALL by regulating the CBL-mediated IRF4/CDK6 axis (Cbl proto-oncogene protein and Interferon regulatory factor 4/cyclin-dependent kinase 6, respectively; by inhibiting CBL, ubiquitination of IRF4 and thus inhibition of CDK6) [25]. Furthermore, miR-155 seems to promote ALL cell proliferation by targeting *ZBTB18* (also known as *ZNF238*; encoding zinc finger and BTB domain-containing 18 protein), whereas anti-miR-155 treatment seems to prevent CD154-mediated repression (i.e., CD40 ligand) of BCL2 and to reduce proliferation in leukemic cells in vitro [27,110]. Extremely high expression of miR-155 has been associated with poor prognosis, whereas in vitro experiments have shown that anti-miR-155 administration seems to be regulatory for ALL cell viability, proliferation, cell cycle and apoptosis; this antileukemic effect has been reinforced by concomitant prednisolone treatment [44,154]. High miR-155 levels were correlated with *KMT2A*-r cases, although miR-155 expression is not essential for leukemia development, so treatment with antagomirs in the respective cases would probably fail [155].

#### 3.3.3. miR-181 Family

Significant upregulation of miR-181 family members has been associated with ALL cases compared with healthy children [10,27,28,35,46,58,69,74,76,83,84,86]. This miRNA family consists of six members: miR-181a-1 clustered with miR-181b-1 in chromosome 1, miR-181a-2 clustered with miR-181b-2 in chromosome 9 and miR-181c clustered with miR-181d in chromosome 19 [8].

Significantly reduced levels of miR-181a-1, miR-181c and miR-181d in *ETV6*-*RUNX1*-positive cases compared with other pediatric BCP-ALL cases can be explained by the fact that this fusion gene and *MIR181A1* regulate each other, whereas a double-negative loop involving these two genes seems to contribute to *ETV6*-*RUNX1*-driven arrest of differentiation in pre-B ALL [153]. In vitro studies in ALL cell lines have demonstrated that treatment with miR-181a induces a significant antileukemic effect against the *ETV6*-*RUNX1* fusion gene [156]. Besides its role as an oncomir, miR-181a also seems to be involved in GC response. Anti-miR-181a treatment of ALL cells has achieved favorable results with respect to proliferation and apoptosis rates, whereas this effect was augmented by prednisolone administration [154]. Expression levels of this miRNA family are associated with pediatric *KMT2A*-r cases (specifically with miR-181b), whereas specimens with deregulated *TLX1* seem to be the only case of significantly low miR-181d at diagnosis compared to healthy individuals [76,79]. Inhibition of miR-181a expression in ALL cells revealed an influence on CD4 and HMGB1 (high-mobility group box 1 protein) levels, indicating a possible role of miR-181a in immunogenicity [157]. Expression levels of miR-181a and miR-181b have been negatively correlated with target genes involved in immune response (*IL1RN*, *BCL6*, *CCR1*, *PTAFR*, *FN1*, *CCL2*, *TLR5*, *TLR8*, *LTB4R*, *IL6R*, and *TNFSF1* or *LTA* encoding lymphotoxin alpha), implying their role in escaping immune surveillance [158]. The interplay between miR-181b-1 and NF-κB was recently described as an amplifying loop linking inflammation to cancer [159]. STAT3 is not only a downstream target of IL-6, but, along with miR-181b-1, miR-21, PTEN and CYLD, lysine 63 deubiquitinase is part of the positive feedback loop regulating the epigenetic switch linking inflammation to cancer [160]. In addition, miR-181a overexpression in T-ALL cells seems to downregulate *EGR1* expression (early growth response 1 zinc finger transcription factor), thereby increasing cell proliferation and enhancing cell cycle progression from the G1 to S phase [161]. Bioinformatics analysis in B-ALL samples revealed that miR-181c regulates lncRNA XIST (X inactive-specific transcript) and that miR-181a-2 and miR-181b-2 are regulated by transcription factors including CDX2 (caudal-type homeobox 2) [162]. miR-181 family levels have been associated with OS (overall survival) in ALL [163]. In the same context, miR-181b and miR-708 levels seem to be significantly higher in high-risk common-ALL compared with standard- and intermediate-risk common-ALL [74].

#### 3.3.4. miR-708

Significant upregulation of miR-708 has been documented in children with ALL compared with controls [27,28,66,74,83]. Despite the latter fact, *BCR*-*ABL1*-positive and *KMT2A*-r pediatric cases seem to have significantly lower miR-708 levels compared with other pediatric ALL cases [29,81,83,164]. Most importantly, lower miR-708 levels seem to efficiently discriminate pediatric T-ALL from B-ALL cases more accurately than any other miRNA [29,66,81,118,131,164,165]. Low expression of miR-708 has been also associated with *PAX5* deletion, whereas high miR-708 expression has been correlated with *ETV6* deletion [131]. As mentioned above, significantly higher miR-708 values have been found in children with high-risk common-ALL, whereas miR-708 seems to target the 3΄-UTR of *CNTFR*, *NNAT* and *GNG12* (encoding ciliary neurotrophic factor receptor, neuronatin and G protein subunit gamma 12, respectively) [74]. Beyond identification of ALL cases, miR-708 levels can be utilized in disease risk stratification and prediction of GC response [112,131,164].

#### 3.3.5. Other Upregulated miRNAs

Significant elevation of miR-130b levels has been documented in children with ALL compared with controls [10,28,84,86]. This miRNA gene resides in chromosome 22 and is clustered with miR-301b, whereas their miRNA family comprises two more clustered miRNAs, miR-130a and miR-301a [8]. Complementary to these reports, it is also known that pediatric *ETV6*-*RUNX1*- and *BCR*-*ABL1*-positive cases have significantly lower levels of miR-130b compared with other BCP-ALL cases [100,153]. Apparently, downregulation of miR-130a and miR-130b in BCR-ABL1 kinase presence is chaperoned by a decrease in CCN3 (cellular communication network factor 3), a negative growth regulator [166].

Upregulation of miR-222 seems to be another trait of pediatric ALL cases when compared with healthy children [36,46,69,77,85]. This miRNA is clustered with miR-221 in chromosome X [8]. Both of these miRNAs seem to target cyclin-dependent kinase inhibitors CDKN1B and CDKN1C (also known as p27 and p57, respectively), and when growth factors are scarce, they lead to quiescence bypass and induce precocious S-phase entry [167]. PTEN is a target of miR-221 and miR-222, and their levels can affect radiation sensitivity via the PTEN/AKT pathway [168]. Similarly, treatment with anti-miR-222 inhibitor has resulted an in vitro decrease in cell proliferation, migration, tube formation and induced apoptosis by upregulation of *ARID1A* (AT-Rich Interaction Domain 1A) and *PTEN* [169]. Downregulation of the *ETS1* proto-oncogene is a hallmark of ETP-ALL (early T-cell precursor ALL) and is associated with the miR-221~222 cluster. Significant elevation of this cluster seems to efficiently discriminate ETP-ALL from non-ETP T-ALL cases, whereas in vitro studies have shown that miR-222 seems to regulate proliferation, cell cycle and apoptosis in leukemia cells [170]. Another upregulated miRNA in pediatric ALL is miR-210. Significantly elevated expression of miR-210 compared with healthy children has been documented in many studies [10,27,38,74,86]. Among pediatric BCP-ALL cases, the lowest values of miR-210 are found in *BCR*-*ABL1*-positive cases [29]. Low miR-210 expression has been associated with poor prognosis and resistance to treatment [171]. Interestingly, an hypoxic environment leads to excessive miR-210 upregulation; therefore, it is considered the miRNA of hypoxia. Other roles of miR-210 include (i) immune response: negative feedback regulator in lipopolysaccharide/toll-like receptor 4 (LPS/TLR4) signaling; (ii) transformation, leukemogenesis and chemotactic invasion: positive feedback loop with NF-κB, IL-6 and STAT3; (ii) DNA repair of double-stranded breaks in cases of hypoxia-induced genomic instability by targeting RAD52; and (iv) activation of the PI3K/AKT pathway by targeting SHIP1 (also known as INPP5 inositol polyphosphate-5-phosphatase D) has been correlated with MDS (myelodysplastic syndrome) development [172].

Significant elevation of miR-363 levels seems to be a unique trait of children with ALL when compared with controls [10,27,69,84,86]. This miRNA is clustered with miR-18b, miR-19b-2, miR-20b, miR-92a-2 and miR-106a in chromosome X [8]. Although studies regarding miR-363 levels in pediatric ALL are lacking, network analysis was recently used to decipher the interplay between this miRNA and NOTCH1 as a pathogenetic marker for cancer development [173]. Another miRNA originating from chromosome X, miR-506, has been associated with childhood ALL occurrence. This miRNA is clustered with miR-507, miR-508 and miR-513a-2 and belongs to the miR-506~514b family, which comprises 18 members [8]. A recent large case–control study reported significantly higher expression levels of serum miR-506 and miR-922 in children with ALL than in controls [31]. However, this finding might be explained in part by the extremely high levels of both of these miRNAs in pediatric *ETV6*-*RUNX1*-positive and *KMT2A*-r cases, corresponding to one-fifth of total pediatric ALL cases [31,174].

Circulating levels of miR-21 seem to be a cancer biomarker according to a relevant meta-analysis. In particular, individuals with high levels of miR-21 in plasma or serum have a 17-fold increased chance of being diagnosed with cancer [175]. Significantly elevated expression of miR-21 in children with ALL has been reported in four studies [30,36,46,60], whereas two studies have reported otherwise [56,68]. Known targets of miR-21 include SERPINB5 (also known as maspin) and PTEN, along with tumor suppressors TPM1 (tropomyosin 1) and PDCD4 (programmed cell death 4 protein) [175]. Moreover, miR-21 downregulation seems to decrease cellular viability and proliferation, whereas targeting of miR-21 results in autophagy induction and sensitivity of leukemic cells to etoposide and doxorubicin [176]. Inhibition of miR-21 has been also been associated with enhancement of daunorubicin toxicity against leukemia cells mediated by PTEN upregulation and the subsequent suppression of the PI3K/AKT pathway [177,178]. With respect to T-ALL development, two pathogenetic links have been described to date: (a) the signaling axis involving miR-21 and PDCD4 in Notch-mediated induction of T-ALL and (b) miR-21-mediated STAT3 inhibition, prompting increased proliferation and invasion and diminishing the apoptosis rate [179,180]. Recently, a study on children with B-ALL correlated upregulation of miR-21 with poor response to induction therapy, as well as shorter DFS (disease-free survival) and OS (overall survival) [60].

Two other important miRNAs in childhood ALL are miR-145 and miR-146a, both originating from chromosome 5. Significant higher levels of miR-145 in children with ALL compared with healthy individuals have been reported three times in the literature [27,28,36]. On the contrary, one study reported low levels of miR-145 in a pediatric ALL population [86]. Significant decreases in miR-145 have also been reported in children with T-ALL compared to controls [10,28]. As mentioned above, cancer development is associated with eradication of the miR-143~145 cluster [144]. A mouse model demonstrated that loss of miR-145 results in leukemia, whereas another study has positively correlated miR-145 levels with the expression of proapoptotic genes *PRKAA1*, *PTEN*, *FOXO3* (encoding Forkhead Box O3 protein), *BCL2L11* (aka BIM), *DIDO1* (encoding death inducer-obliterator 1) and *BCL2L13* [158,181]. Cell cycle, proliferation, apoptosis and invasion are among the cellular processes that miR-145, as a potent tumor suppressor, regulates, whereas p53 has been shown to induce miR-145 levels, which, in turn, inhibits MYC and MUC1 (mucin 1 cell surface-associated protein) [182,183]. The complex interplay between leukemic cells and the microenvironment (along with methodological discrepancies) may explain the contradictory results with respect to the miR-143~145 cluster in childhood ALL [184]. Published data regarding miR-146a are far more transparent [27,28,33,36,46,48,65,69,77,86]. Upregulation of miR-146a seems to be ubiquitous across childhood ALL, with the exception of the *TCF3*-r BCP-ALL subtype [29,100]. Two studies that have reported otherwise are disputable (very small sample size and not significant results, respectively) [40,47]. As analyzed in the corresponding section, miR-146a expression levels may vary depending upon polymorphisms [47,89]. This miRNA seems to promote cell proliferation, migration and invasion, in addition to inhibiting cell apoptosis by downregulating its target, *CNTFR* (ciliary neurotrophic factor receptor), whereas low *CNTFR* expression leads to LIF inhibition (LIF interleukin 6 family cytokine) and JAK2/STAT3 pathway activation [48]. High expression of miR-146a is most prevalent among biphenotypic ALL patients, whereas some evidence associates this miRNA with WBC at diagnosis [65]. Furthermore, miR-146a plasma levels in children with poor-prognosis ALL seem to be significantly higher than in children with favorably prognostic characteristics; this finding may be attributed to the fact that miR-146a interferes with prednisolone efficacy [121,154]. Besides its major role in T-ALL, miR-146a seems to be implicated in B-cell maturation through miR-the 146a/NFKB1/BCL11A pathway and in megakaryopoiesis regulated by the ZBTB16/miR-146a/CXCR4 axis (ZBTB16 or zinc finger and BTB domain-containing 16 protein alias PLZF of ZNF145; C-X-C motif chemokine receptor 4 alias CD184 or fusin) [185,186]. This miRNA has been also used to discriminate ALL from AML cases, but in both cases, miR-146a seems to confer poor OS [163].

### 3.4. miRNAs and ALL Subtypes

We investigated the literature for differentially expressed miRNAs across childhood ALL subtypes and found considerable heterogeneity among studies, even in terms of defining subgroups within ALL. A review of published data (Supplementary Table S1) revealed the involvement of several miRNAs in T-ALL development (reported by ≥2 studies), i.e., childhood T-ALL versus controls has been associated with upregulation of miR-16, miR-19b, miR-92a, miR-130b, miR-146a, miR-181a, miR-181b and miR-221 and with downregulation of miR-29a, miR-145 and miR-574.

To verify the importance of specific miRNAs in leukemogenesis, we reanalyzed the only available small RNA-seq dataset of pediatric T-ALL cases compared with controls (Supplementary Table S2) [10]: hsa-miR-16-5p (rank #57; *p* = 0.0012), hsa-miR-19b-3p (#90; *p* = 0.011), hsa-miR-92a-2-5p (#48; *p* < 0.001), hsa-miR-130b-3p and -5p (#14 and #127; *p* < 0.001 and *p* = 0.04, respectively), hsa-miR-181a-5p, -2-3p and -3p (#15, #18 and #29; *p* < 0.001), hsa-miR-181b-5p and -3p (#2 and #25; *p* < 0.001), hsa-miR-145-5p (#20; *p* < 0.001) and hsa-miR-574-3p (#9; *p* < 0.001). Adjusted *p* values for hsa-miR-221-3p and -5p, hsa-miR-29a-3p and hsa-miR-146a-5p did not reach the significance level. Interestingly, analysis of effect size estimates highlighted the importance of differentially expressed hsa-miR-574-3p, hsa-miR-618, hsa-miR-504-5p, hsa-miR-4488, hsa-miR-6500-3p, hsa-miR-153-3p, hsa-miR-548a-3p, hsa-miR-2682-5p, hsa-miR-466, hsa-miR-4492, hsa-miR-106a-3p, hsa-miR-3196, hsa-miR-135a-5p and hsa-miR-1281 (log_2_ fold change > 5) in children with T-ALL compared with controls. hsa-miR-128-3p ranked first among differentially expressed miRNAs (lowest adjusted *p* value).

Downregulation of miR-16 has been long known to be a main event in CLL (chronic lymphocytic leukemia), but its upregulation seems to play a pivotal role in regulating BCL2 (by binding the 3′-UTR region of *BCL2*) and in mediating prednisolone-induced apoptosis in childhood T-ALL [79,187,188,189,190]. In addition, miR-16-1 levels have been proposed as a prognostic biomarker for OS, although discrepancies do exist [187,190]. Some BCL2 inhibitors (venetoclax or ABT-199, navitoclax or ABT-263 and ABT-737) have achieved favorable outcomes in specific pediatric ALL groups, especially in ETP-ALL, hypodiploid B-ALL and BCR-ABL1-positive cases; this confirms a major role for BCL2 and the miR-16 precursor family (miR-15a, miR-15b, miR-16-1, miR-16-2, miR-195 and miR-497) in leukemogenesis and apoptosis [191,192].

The miR-17~92 cluster in chromosome 13, a polycistronic family comprising miR-17, miR-18a, miR-19a, miR-19b-1, miR-20a and miR-92a-1, is implicated in many hematological cancers. In specific, miR-19 has been proven sufficient to promote leukemogenesis in Notch1-induced T-ALL in vivo and to influence cell cycle progression by targeting cyclin-dependent kinase inhibitor *CDKN1A* (p21), whereas inhibition of miR-19b seems to lead Jurkat cells to increased apoptosis [193,194]. Interestingly, miR-19 targets BIM (BCL2-like 11 protein) encoded by *BCL2L11*, *PRKAA1* (encoding protein kinase AMP-activated catalytic subunit alpha 1), *PTEN* (encoding phosphatase and tensin homolog) and *PPP2R5E* (encoding protein phosphatase 2 regulatory subunit B’epsilon), whereas miR-92 regulates *FBX7* (encoding F-box protein 7), *BCL2L11*, *IKZF1* (encoding IKAROS family zinc finger 1 protein), *PTEN* and *NF1* (encoding Neurofibromin 1), indicating that regulators of PI3Ks are crucial for childhood T-ALL [79,193]. Moreover, a recurrent gain of 13q31.2-q31.2 in the aforementioned cluster’s region has been noted as a common finding (14%) in BM samples of untreated pediatric patients with T-ALL, resulting in increased miR-19 expression levels [194]. Oncomir properties of miR-19b and miR-92a in childhood T-ALL have been substantiated by case–control studies [20,79].

Upregulation of 130b has been implicated in T-ALL occurrence, especially in *TAL*-r T-ALL pediatric cases [10,28]. This miRNA has been associated with drug resistance, and it is an essential driver of *KMT2A*-*AFF1* leukemia, integrated with the aberrant overexpression of key downstream targets, i.e., the antiapoptotic factor BCL2 and proto-oncogene *MYC* [195,196]. The role of miR-146a in childhood ALL is discussed above in the text.

Overexpression of clustered miR-181a and miR-181b seems to be universal for childhood ALL, except for *ETV6*-*RUNX1*-positive cases [10,28,153]. miR-181a has been implicated in T-ALL development and has also exhibited regulatory effects towards the development and differentiation of B-cells and cytotoxic T-cells by diminishing the expression of genes involved in thymocyte maturation (such as *BCL2*, *CD69* and those of TCR) [28]. Silencing of exosomal miR-181a reverses childhood ALL cell proliferation in vitro by upregulating proliferative (*PCNA*-encoding proliferating cell nuclear antigen and *MKI67*-encoding marker of proliferation Ki-67) and pro-survival genes (*MCL1* apoptosis regulator and *BCL2*) and by suppressing proapoptotic genes (*BAD* of BCL2-associated agonist of cell death and *BAX* of BCL2-associated X apoptosis regulator) [197]. With respect to miR-181b, its upregulation in childhood BCP-ALL specimens has been associated with methylation of the promoter regions and subsequent downregulation of tumor suppressor genes *PPP1CA*, *BTG2* and *PTEN*, and these effects have been restored with decitabine treatment in vitro [108]. Among others, ALL-related targets of miR-181a and miR-181b include ERK/MAPK, mTOR and NF-κB signaling pathways, whereas the expression of this cluster seems to be abundant in the early B-cell stage and to decline progressively with maturation [198].

Significant overexpression of miR-221 was associated with T-ALL diagnosis in two case–control studies [33,73]. High levels of both miR-221 and miR-146a seem to accurately discriminate T-ALL pediatric cases from healthy controls [33]. This miRNA is embroiled in hematopoietic stem cell differentiation and DNA damage response, and it is known to downregulate *CDKN1B* [148]. An in vitro study reported that miR-221 may exert inhibitory effects towards bone marrow mononuclear cells and their abilities to proliferate, migrate and invade in childhood ALL cases, all by inhibiting the Wnt/β-catenin signaling pathway, as demonstrated by reduced PCNA (proliferating cell nuclear antigen), cyclin D1 and MMP9 (matrix metallopeptidase 9). In the context of previous reports, miR-221 presence has been linked with a significant increase in cells in the G0/G1 phase and an increase in apoptotic cells accompanied by increased caspase 3 levels [199]. Overexpression of miR-221 concerns solely T-ALL (and AML), whereas there are indications that it is downregulated in B-ALL (especially in *ETV6*-*RUNX1* and *KMT2A*-r cases) [29,69,100,148,153,200]. In addition, children with T-ALL/CD56^+^ seem to have significantly higher miR-221 levels compared with T-ALL/CD56- patients (271.4-fold; CD56 or NCAM1 i.e., neural cell adhesion molecule 1 expression confers poor prognosis in T-ALL), and pediatric immature T-ALL cases have significantly higher miR-221 levels than all other T-ALL subgroups [20,73].

Low expression of miR-29a in pediatric T-ALL has been documented in two studies [71,79]. In general, miR-29a acts as a tumor suppressor and affects the methylation status of certain target genes, thus contributing to T-ALL pathogenesis [71]. This miRNA, together with miR-7 and miR-195,s seem to play a crucial role in pediatric T-ALL, invariably downregulated in most T-ALL subgroups: *TLX1*-, *TLX3*- and *TAL1*-deregulated, *PICALM*-*MLLT10* and inv(7) cases [79]. Expression of miR-29a among pediatric B-ALL cases varies significantly (high levels in *BCR*-*ABL1* cases and low levels in *TCF3*-r and *KMT2A*-r cases) [29,62,69,79,100]. Leukemia patients with *TP53* abnormalities display deregulated miR-29 family expression profiles, and downregulation of miR-29c has been associated with upregulation of *MCL1* and *TCL1* (encoding TCL1 family AKT coactivator A) proto-oncogenes [201]. Significantly lower levels of both miR-145 and miR-574 have been reported in children with T-ALL compared to controls in two independent studies [10,28]. Low miR-574 expression seems to efficiently discriminate childhood T-ALL from B-ALL patients, whereas *TLX1*-deregulated cases seem to be excluded from this pattern [79,165]. Notably, significantly higher levels of miR-574 have been found in BCP-ALL, *KMT2A*-r and *TCF3*-*PBX1* subtypes in comparison with other B-ALL cases [202].

Investigation of differentially expressed miRNAs in T-ALL subgroups (compared with controls, ALL or T-ALL samples) revealed a prevalent miRNA signature across pediatric T-ALL subgroups: upregulation of miR-146a, miR-182, miR-196b, miR-223, miR-376a and miR-662,and downregulation of miR-7, miR-29a, miR-99a, miR-195, miR-296 and miR-422a [20,48,79,106,116]. The aforementioned signature of T-ALL subgroups is in agreement with a literature review regarding T-ALL case–control studies in the context of miR-146a upregulation and miR-29a downregulation. However, reanalysis of the small RNA-seq dataset failed to confirm the significant differential expression of miR-146a and miR-29a. Further large cohort studies are needed to confirm our results.

Further analysis of miRNA signatures in other childhood ALL subgroups is beyond the scope of this review and is covered in Supplementary Table S1. A recent meta-analysis of differentially expressed miRNAs in B-ALL compared with controls across 25 studies (including both adult and pediatric samples) revealed no consensus miRNA signature. Eight miRNAs in the latter meta-analysis showed promising insight with respect to diagnosing B-ALL: upregulation of miR-128-1, miR-128-2, miR-142, miR-155, miR-181a, miR-181b and miR-181c and downregulation of miR-451a [198]. Our review of miRNA levels in pediatric B-ALL compared with controls revealed that miR-155 is the most frequently reported upregulated miRNA (followed by miR-146a, miR-181b, miR-222 and miR-708; two citations for miR-16, miR-21, miR-34a, miR-100, miR-128-1, miR-181a, miR-181c, miR-195, miR-210, miR-320a and miR-660), whereas the most frequently reported downregulated miRNA is miR-374a (two citations for miR-27a, miR-30c, miR-196b, miR-223 and miR-494). With respect to miR-374a, it is clustered with miR-545 at the X-chromosome inactivation center and seems to regulate cell growth and differentiation, although there are inconsistent reports in literature [203].

MicroRNAs have also been employed to discriminate pediatric T-ALL from B-ALL cases, and differential expression analysis designated miR-196b as the top upregulated miRNA (followed by miR-542) and miR-708 as the top downregulated miRNA (followed by miR-151a, miR-425 and miR-497; Supplementary Table S1).

## 4. Discussion

**Figure 4:**
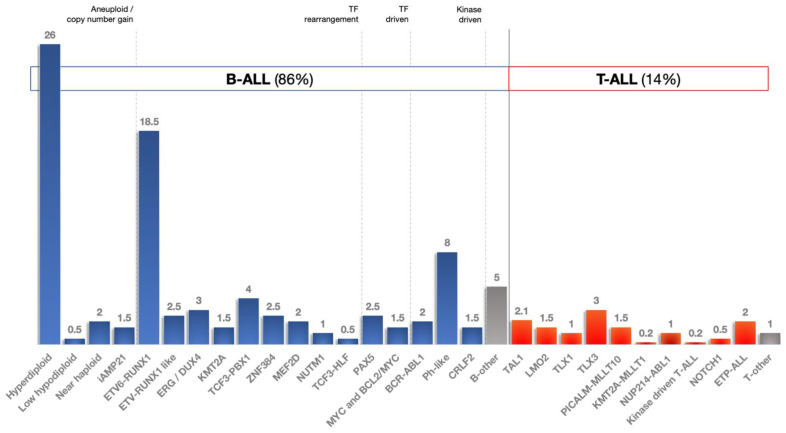
Childhood ALL subtypes.

Most of case–control studies that have been carried out regarding childhood ALL have traits that limit reproducibility and increase bias. The main flaws in methodology that have been determined can be categorized in seven tiers:

[i] Sample issues: Sixteen publications in this systematic review had considerably small sample sizes (30 or less; 26%) [36,40,41,46,48,49,58,64,68,69,77,78,80,82,84,86]. Larger sample sizes are required to achieve sufficient statistical power and allow for solid conclusions to be drawn. Another problem is the small number of healthy participants in case–control studies; 76% of included studies had 30 or fewer controls (Table 1). The proportion between the number of children with ALL and controls in also of interest and may affect measured expression levels and outcomes. In seven studies, participating healthy controls accounted for 10% or less of children with ALL (2.2%, lowest ratio) [35,53,54,66,75,76,83]. In one case, controls were adults and not age-matched [69], whereas in four studies, preserved cells or cell subsets obtained by donors of unknown age and health status were used [66,78,79,82]. One qRT-PCR from the Netherlands utilized CD34^+^ progenitor cells from two children with brain tumor as controls, and one another study used cancer-free children with fractures as controls, although it is unclear whether these practices could interfere with measured outcomes [59,83]. It is common sense and in line with reports that associate miRNA levels with tumorigenesis and inflammation that only samples from completely healthy children (age-matched if applicable) should be obtained as controls. Another issue regarding samples that might influence miRNA expression measurements is the proportion of each ALL subtype within each sample. Figure 4 represents our current knowledge on representation of each subtype within childhood ALL [1,2,3,4,5]. For example, it is erroneous to include more *KMT2A*-r cases of T-ALL in the same ALL sample; instead, separate calculations should be employed. Weighted samples in terms of cytogenetics and subtype classification should be always considered, excluding cases investigating specific subtypes alone [67,72,83]. Previous research in the field has shown that population characteristics (ethnicity, race, etc.) may also play a role in childhood ALL susceptibility, especially when studying the effects of specific SNPs on the disease.

[ii] Origin of samples: Expression of miRNAs has been demonstrated to be tissue-specific. Heterogeneity of results could be explained to some extent by measurements in different specimens: BM, PBMCs, serum or plasma. Simultaneous study of miRNA expression patterns in both BM and peripheral blood has resulted in contradictory results; one study reported no significant difference, whereas two other studies reported significant fluctuations [28,46,68]. Accordingly, a qRT-PCR study on adults with ALL reported significantly lower levels of oncomiR miR-92a in fresh leukemia cells compared with CD34^+^ cells obtained from healthy volunteers (*p* = 0.0014), significantly lower expression of miR-92a in ALL subjects compared to controls (*p* = 0.001) and a significantly higher cell-to-plasma ratio of miR-92a in ALL cells (*p* < 0.0001) compared with those in peripheral blood specimens obtained from healthy individuals, indicating that leukemia cells retain miR-92a [204]. Circulating miRNAs in serum and plasma show significant stability, rendering them reliable biomarkers for discrimination of childhood ALL cases from healthy children [24].

[iii] Time point in therapy: A sample size comprising children with ALL in different stages of therapy is not appropriate and may tamper miRNA expression profiles. In terms of prognosis and drug sensitivity, study of matched samples before and after therapy is strongly encouraged. Newly diagnosed children with ALL, as well as relapsed and remitted cases, were detected together in five case–control studies included in the present review [32,41,43,46,54].

[iv] Handling of outliers: With respect to children from distinct cytogenetic groups, it is essential to keep the respective proportions within the ALL group or study subgroups individually, as stated above. Another problem is cases with high leukemic blast burden; therefore, predetermined cutoff values are recommended, and enrichment should be applied in cases with inadequate blasts [24].

[v] Methods and validation: The most commonly used method to determine expression levels of miRNAs is qRT-PCR followed by microarrays (Table 1). Nevertheless, RNA-seq and other NGS (next-generation sequencing) methods with revolutionize miRNA detection and quantification in the coming years. Substantial technical requirements, extensive amplification, time and cost are the main disadvantages of these sequencing methods, but identification of isomiRs and novel miRNAs are in the armamentarium of RNA-seq. Poor primer and probe design, along with selection of inappropriate normalization controls, is among the most common flaws in qRT-PCR. Validation of NGS results with RT-PCR is considered a gold standard [205].

[vi] Reproducibility: Data of future studies must be transparent and reproducible in subsequent studies. The data of most large-scale studies included in this review are not deposited in GEO (Gene Expression Omnibus) or similar platforms.

Various miRNA SNPs seem to be associated with predisposition to childhood ALL in the literature. The systematic review of the literature and meta-analysis performed in the present study on all relevant data revealed significant protective roles of SNPs in pri-miR-34b/c and miR-100, whereas predisposition to B-ALL was associated with SNPs in *DROSHA*, miR-449b, miR-938, miR-3117 and miR-3689d-2 genes (Figure 2). Variant allele rs1573613 T>C of *ETV6* seems to weaken the binding of miR-34c-5p and miR-449b-5p (resulting in 17% and 33% increase protein levels relative to the T allele, respectively), whereas these interactions double the risk for childhood ALL (OR, 1.9; 95% CI: 1.16 to 3.11; *p* < 0.05) [206]. The expression of miR-34b/c in childhood ALL is inhibited by methylation of its promoter, which in turn impairs the restraining effects of miR-34b on cell proliferation and response to prednisone [63,120]. Moreover, miR-34b/c seems to be a tumor suppressor (part of the p53 network), and the C allele of rs4938723 in pri-miR-34b/c confers increased transcriptional activity of the miR-34b/c promoter [98]. The AA genotype of rs12402181 in miR-3117-3p affects the seed region of miR-3117-3p, whereas the CT/CC genotype in rs62571442 of miR-3689d-2 leads to changes in the respective pre-miRNA. Both of these SNPs lead to energy changes, alterations in secondary miRNA structure and aberrant activation of the MAPK/ERK pathway [94]. Although studies are lacking, miR-3117 seems to target *CD274* (alias PD-L1 or programmed cell death 1 ligand 1 gene), and miR-3689d-2 targets *CAPN6* (encoding calpain 6), which is implicated in the NF-κB and B-cell receptor signaling pathways [8,207]. The role of miR-100 in childhood ALL was elaborated earlier in the text.

Evaluation of published data on differential expression of miRNAs in children with ALL compared with controls generated some interesting conclusions: (i) there are substantial differences in study designs of published material, making it impossible to conduct an unbiased meta-analysis; (ii) upregulation of the miR-128 family, miR-130 family, miR-155, miR-181 family, miR-210, miR-222, miR-363 and miR-708, along with downregulation of miR-143 and miR-148a, seems to play a definite role in childhood ALL development; (iii) other important miRNAs involved in ALL pathogenesis in children include differentially expressed let-7e, let-7f, miR-24, miR-99a, miR-100, miR-125b, miR-145, miR-146a, miR-182, miR-196b, miR-199b, miR-223 and miR-335; and (iv) significant upregulation of miR-130b, miR-181a and miR-181b and downregulation of miR-145 and miR-574 were confirmed by our re-analysis as putative biomarkers of T-ALL presence in childhood.

The performed reanalysis of small RNA-seq data derived from pediatric T-ALL cases and controls revealed a top-10 signature that could be utilized for diagnostic purposes in the future: (i & ii) hsa-miR-128-3p and hsa-miR-181b-5p are major regulators of early lymphoid differentiation that prevent stem-progenitor cells from maturation and contribute to escape from immune surveillance [46,158,159]; (iii) hsa-miR-130a-3p induce modulation of cell survival programs by regulating autophagic flux and inhibition of *DICER1*, a major component of RNA-induced silencing complex (RISC) [208]; (iv & vi) hsa-miR-106a-5p and hsa-miR-20b-5p, clustered together, are members of the miR-17 family and major drivers of tumorigenesis in various cancers [144]; (v) hsa-miR-30a-5p, the downregulation of which results in apoptosis inhibition, promotes proliferation and stimulates migration and invasion through PI3K/AKT/mTOR and miR-30a/NOTCH1/MYC pathways [209,210]; (vii) hsa-miR-24-3p, the downregulation of which has been associated with *PAX5* deletion, whereas its deregulated levels interfere with the expression of both *MYC*, *BCL2* and *HIF1A* (hypoxia-inducible factor 1 subunit alpha) oncogenes and p21 and p53 tumor suppressor proteins [131,211]; (viii) hsa-miR-143-3p induced the deletion of the miR-143~145 cluster has been designated as a hallmark of tumorigenesis in various cancers [144]; (ix) has-miR-574-3p, the reduced expression of which seems to promote proliferation and inhibit apoptosis in leukemic cells by targeting the IL6/JAK/STAT3 pathway [212]; (x) hsa-miR-618, the downregulation of which has been previously reported in childhood ALL, in addition to being implicated in lymphoma pathogenesis via p53, *STAT3*, *HDAC3* (histone deacetylase 3), *CUL4A* (cullin 4A) and *FKBP3* [10,27,213].

Recently, ALLSorts, a method that utilizes RNA-seq data to classify pediatric B-ALL samples into 18 known subtypes and 5 meta-subtypes, was made publicly available [214]. Subtype-specific miRNA signatures bear resemblance with those previously published in the literature (Supplementary Table S1; Figure 4). RNAseqCNV is another tool that can detect large-scale CNVs (copy number variations) from RNA-seq data. This method is consistent with DNA-based techniques and more effective than conventional cytogenetic studies in determining ALL subtypes [215].

## 5. Conclusions

In conclusion, many miRNA case–control studies in the field of childhood ALL suffer from high risk of bias and limitations of evidence, including inconsistencies in methodology and study designs that preclude reproducibility and proper meta-analysis. Larger cohort studies and utilization of novel NGS tools will revolutionize childhood ALL diagnosis and subtype classification henceforth.

## Figures and Tables

**Table 1:** Childhood ALL diagnosis and miRNAs.

Study	Cohort Characteristics	Methods	Outcomes
Sun et al., 2021 [25]	43 childhood ALL patients and 14 controls; validation: 28 childhood ALL patients and 28 controls; China	qRT-PCR and microarray; BM	Significant upregulation of **miR-155**-5p in ALL samples compared with controls (*p* < 0.05).
Zamani et al., 2021 [26]	59 children with ALL at diagnosis and 50 non-cancer controls; Iran	qRT-PCR; BM	Significantly lower **miR-324-3p** and **miR-508-5p** expression in children with ALL (*p* < 0.0001 and *p* < 0.005, respectively). ROC analysis evaluated the utilization of miR-324-3p (AUC = 0.73; sensitivity, 44% (95% CI: 29.99 to 58.75); specificity, 100% (95% CI: 92.75 to 100); cutoff value, 0.9506; *p* < 0.0001) and miR-508-5p (AUC = 0.664; sensitivity, 40%; 95% CI: 26.41 to 54.82; specificity, 95.92%; 95% CI: 86.02 to 99.5; cutoff value, 0.1812; *p* = 0.005) as potential diagnostic biomarkers in pediatric ALL.
Liang et al., 2021 [27]	42 childhood ALL patients at diagnosis and controls from GEO databases GSE56489 (43 children with ALL and 14 controls) [28] and GSE23024 (81 children with ALL and 7 controls) [29]; China	qRT-PCR and microarray; BM	Differentially expressed miR-155 and miR-199b were documented in all three datasets. Analysis of datasets showed several other differentially expressed miRNAs. With respect to the GSE56489 dataset, significant differential expression of **miR-606**, **miR-640**, **miR-199b**-3p, **miR-145**, **miR-297**, miR-181a*, **miR-1322**, **miR-155**, **miR-146a**, **miR-587**, **miR-1323**, let-7b*, **miR-548i**, **miR-3121**, **miR-449a**, **miR-369**-3p, **miR-708** and **miR-181b** was observed in ALL patients compared to controls (*p* < 0.05). With respect to the GSE23024 dataset, significant differential expression of **miR-199b**, **miR-432**, **miR-224**, **miR-376a**, **miR-148a**, **miR-485**-3p, **miR-411**, **miR-382**, **miR-503**, **miR-223**, **miR-199a**, **miR-450**, **miR-338**, **miR-424**, **miR-145**, **miR-143**, **miR-618**, **miR-128b**, **miR-363**, **miR-497**, **miR-153**, **miR-659**, **miR-34a**, **miR-181b**, **miR-181d**, **miR-181c**, **miR-130a**, **miR-210**, **miR-130b**, **miR-579** and **miR-155** was observed in children with ALL compared with healthy children (*p* < 0.05).
El-maadawy et al., 2021 [30]	43 childhood ALL patients and 42 controls; Egypt	qRT-PCR; PBMCs	Significant elevation in **miR-21** (*p* < 0.05), **miR-148a** (*p* < 0.01) and **miR-24** (*p* < 0.05) expression levels in ALL patients compared to controls. No significant change in miR-26a and miR-133b levels. ROC curve analysis demonstrated the highest AUC for miR-24, followed by miR148a and **miR-133b** (0.785, 0.719 and 0.669, respectively). No significant results with respect to miR-21 and miR-26 ROC curves. At a cutoff value of 2.928, miR-24 showed 72% sensitivity and 81% specificity (*p* < 0.001) in detecting ALL cases. Compared to healthy subjects, ALL patients showed a positive correlation between miR-148a and miR-24 (r = 0.347; *p* < 0.05). Another positive correlation was found between miR-26a and miR-24 (r = 0.353; *p* < 0.05) in the ALL group compared to the control group.
Zhu et al., 2021 [31]	132 childhood ALL patients and 80 controls; China	qRT-PCR; serum	Significantly higher expression levels of serum **miR-922** and **miR-506** in children with ALL than controls (*p* < 0.001). ROC curve analysis reported optimal cutoff values of miR-922 and miR-506 for the diagnosis of childhood ALL of 1.46 and 2.17, respectively.
Fayed et al., 2021 [32]	71 childhood ALL patients (32 newly diagnosed, 21 relapsed and 18 remitted) and 30 controls; Egypt	qRT-PCR; plasma	AUC of **miR-92a** (0.755; cutoff 8.77) with 41.5% sensitivity and 100% specificity, whereas **miR-638** showed an AUC of 0.862 and sensitivity and specificity of 54.7% and 100%, respectively (cutoff, 6.79). Levels of miR-92a and miR-638, as well as the miR-92a/miR-638 ratio, were significantly higher (17.89, 10.19 and 1.75-fold, respectively) in children newly diagnosed with ALL compared to controls. There was a significant positive correlation of miR-92a and miR-638 levels in children with ALL (r = 0.955; *p* ˂ 0.0001).
Li Shao-Wu et al., 2020 [33]	130 newly diagnosed children with T-ALL and 50 controls; China	qRT-PCR; PBMCs	Expression of **miR-146a** and **miR-221** in T-ALL subjects was significantly higher than in controls (5.83 ± 1.54 vs. 0.96 ± 0.17 and 7.13 ± 2.6 vs. 1.64 ± 0.51, respectively; *p* < 0.01). Diagnostic cutoff values of miR-146a and miR-221 in childhood T-ALL were determined to be 3.9 and 5.28, respectively, by means of ROC curve analysis. The AUC of T-ALL jointly diagnosed by miR-146a and miR-221 was 0.835 (95% CI: 0.764 to 0.892) with high sensitivity (85%) and specificity (77.2%). In addition, levels of miR-146a were positively correlated with miR-221 levels at diagnosis (r = 0.784, *p* < 0.01).
Li Chunyu et al., 2020 [34]	59 childhood ALL patients at diagnosis and 30 controls; China	qRT-PCR; BM	Expression of **miR-223** was markedly reduced in patients with ALL compared with controls (*p* < 0.001). The ROC curve confirmed the diagnostic value of miR-223 in ALL: AUC, 0.978 ± 0.013; sensitivity, 93.22%; and specificity, 93.33% (cutoff, 0.705; *p* < 0.0001). miR-223 seems to inhibit cell proliferation, migration and invasion and to promote apoptosis by targeting *FOXO1* (which was upregulated in ALL patients).
Shafik et al., 2020 [35]	70 childhood ALL patients at diagnosis and 7 controls; Egypt	qRT-PCR; BM	The expression of **miR-181a** was statistically significantly elevated in ALL patients compared to controls (*p* < 0.001). However, **miR-196b** expression was significantly downregulated in ALL patients compared to controls (*p* = 0.038). High expression of miR-181a was reported in 68 out of 70 ALL patients (97.1%; cutoff, 0.015), whereas 49 of 56 children with ALL (87.5%; cutoff, 0.001) were found to exhibit low miR-196b expression. A significant positive correlation was observed between miR-181a and miR-196b expression levels (r = 0.344; *p* = 0.009).
Akpinar et al., 2020 [36]	13 childhood ALL patients at diagnosis and 5 controls; Turkey	qRT-PCR; whole blood	Significant downregulation of **miR-375** and upregulation of **miR-21**, **miR-222**, **miR-30**, **miR-145**, **miR-146a** and **miR-155** levels in ALL patients compared with controls.
Chen et al., 2020 [37]	42 childhood T-ALL patients (21 primary and 21 recurrent) and 20 controls; China	qRT-PCR; PBMCs	Significant downregulation of **miR-335**-3p in T-ALL patients compared with controls (*p* < 0.05).
N. Hassan et al., 2020 [38]	85 childhood ALL patients at diagnosis and 12 controls; Egypt	qRT-PCR; BM	Expression of **miR-100** was significantly downregulated in ALL patients compared to controls (*p* = 0.035). Expression of **miR-210** was significantly upregulated in ALL patients compared to controls (*p* = 0.025). ROC curve analysis revealed an AUC 0.642 for miR-100 (95% CI: 0.519 to 0.764; cutoff, 2.6; 64.7% sensitivity and 62.5% specificity), whereas the AUC for miR-210 was 0.65 (95% CI: 0.511 to 0.79; cutoff, 3.5; 60% sensitivity and 58.3% specificity).
S.S. Hassan et al., 2020 [39]	60 childhood ALL patients (30 HCV and 30 non-HCV) at diagnosis, 10 controls with HCV and 2 healthy controls; Egypt	qRT-PCR; PBMCs	Hepatitis C virus genotype 4 (HCV-4)-associated ALL cases displayed a 3-fold increase in the expression of **miR-155** compared to children with chronic HCV infection (47.8 vs. 16.2), suggesting it as a therapeutic target in the respective cases. In addition, ALL patients seemed to express higher levels of miR-155, regardless of whether they were HCV-infected or not.
Al Nakeeb et al., 2020 [40]	20 childhood ALL patients and 30 controls; Iraq	qRT-PCR; serum	Expression levels of **miR-142**-3p and **miR-146a**-3p were significantly downregulated, whereas **miR-223**-3p expression was significantly upregulated in ALL patients compared with healthy children.
Dawidowska et al., 2019 [10]	34 childhood T-ALL patients and 5 controls; Poland	RNA-seq and qRT-PCR; BM	Significant overexpression of **miR-548a**-3p, **miR-128**-3p, **miR-181b**-5p, **miR-20b**-5p, **miR-6500**-3p, **miR-331**-5p, **miR-363**-3p, **miR-153**-3p, **miR-466** and **miR-130a**-3p (*p* < 0.01; also miR-20b-3p, miR-210-3p, miR-181a-3p, **miR-4421**, **miR-18b**-5p, miR-181a-2-3p, miR-181a-5p, miR-625-3p, miR-130b-3p, **miR-4687**-5p, miR-4437, miR-625-5p and **miR-3609** with 0.01 ≤ *p* < 0.05) in T-ALL samples compared with controls. Significantly lower levels of **miR-574**-5p, **miR-10a**-5p, **miR-582**-3p, **miR-143**-3p, **miR-941**, **miR-145**-5p, **miR-27a**-5p, **miR-618**, **miR-24-2**-3p, miR-145-3p, **miR-504**-5p, **miR-3690**, **miR-223**-5p, **miR-199b**-5p, **miR-550a**-5p, **miR-4695**-3p, **miR-30a**-5p, **miR-3909**, **miR-2115**-3p, miR-582-5p, miR-504-3p, **miR-23a**-5p, **miR-10b**-5p, **miR-4494** and **miR-151a**-3p (*p* < 0.01; also miR-223-3p, **miR-6865**-3p, **miR-7849**-3p, miR-1275, miR-338-5p, **miR-3150b**-5p, miR-3154, **miR-6823**-5p, miR-10a-3p, miR-143-5p and **miR-4745**-3p with 0.01 < *p* < 0.05) in T-ALL samples compared with controls.
Sheybani et al., 2019 [41]	27 childhood B-ALL patients (non-Ph-positive; 19 at diagnosis and 8 at relapse) and 16 controls; Iran	qRT-PCR; BM	Significant downregulation of **miR-326** was noted in B-ALL patients compared with controls.
Xue et al., 2019 [42]	831 childhood ALL patients and 1079 controls; validation: 88 childhood ALL cases and 99 controls; China	qRT-PCR; whole blood; plasma from validation cohort	Expression levels of **miR-100** in plasma of childhood ALL cases were 3.25-fold higher than in controls (*p* < 0.001).
Pouyanrad et al., 2019 [43]	64 childhood ALL patients (46 at diagnosis and 18 at relapse) and 30 controls; Iran	qRT-PCR; BM	Expression of **miR-335**-3p was found to be significantly downregulated in children with ALL compared with controls (0.33 ± 0.04 vs. 0.7325 ± 0.13; *p* = 0.005). Moreover, miR-335-3p was noticeably downregulated in relapsed patients (0.08 ± 0.02) compared with controls (0.73 ± 0.13; *p* = 0.0002), and miR-335-3p levels were also lower in newly diagnosed ALL patients compared with controls (*p* = 0.018).
El-Khazragy et al., 2019a [44]	45 childhood ALL patients and 10 controls; Egypt	qRT-PCR; BM	Expression levels of **miR-155** and **miR-181a** were significantly higher in ALL patients compared to controls (*p* < 0.01).
El-Khazragy et al., 2019b [45]	120 childhood ALL patients at diagnosis and 30 controls; Egypt	qRT-PCR; PBMCs and BM	Pediatric ALL samples exhibited significantly lower expression of **miR-125b** (*p* < 0.001) compared with controls. In contrast, Bcl-2 expression levels were significantly higher in ALL patients compared with controls (*p* < 0.001). ROC curve analysis illustrated that miR-125b and Bcl-2 expression could be potential biomarkers for discrimination children with ALL from healthy control, with an AUC 0.99, 0.9 reciprocally. However, miR-125b was superior to Bcl-2: miR-125b sensitivity 98.3% and specificity 96.7% (cutoff ≤ 1.5); Bcl-2 sensitivity 96.6% and specificity 70% (cutoff > 2.14).
Rzepiel et al., 2019 [46]	20 childhood BCP-ALL patients (15 at diagnosis and 5 at relapse) and 10 controls; Hungary	qRT-PCR and TLDA; plasma and BM	Various circulating miRNAs in plasma displayed significantly different expressions between BCP-ALL patients and controls: upregulation of **miR-34a**-5p, **miR-128**-3p, **miR-146a**-5p, **miR-155**-5p, **miR-181a**-5p, **miR-181b**-5p, **miR-181c**-5p, **miR-222**-3p, **miR-532**-5p (*p* < 0.001), **miR-21**-5p, **miR-92a**-3p, **miR-125b**-5p, **miR-320a**, **miR-361**-3p, **miR-660**-5p (*p* < 0.01), **miR-16**, **miR-30d**-5p and **miR-93**-5p (*p* < 0.05); downregulation of **miR-223**-3p, **miR-494**-3p (*p* < 0.001) and **miR-374a**-5p (*p* < 0.05). There was no significant correlation between miR expression levels in bone marrow and peripheral blood.
Jemimah Devanandan et al., 2019 [47]	71 childhood ALL patients and 74 controls; India	qRT-PCR; whole blood	Downregulation of miR-146a in ALL patients compared to controls, although not significant.
Wang et al., 2019 [48]	28 childhood ALL patients and 10 controls; China	qRT-PCR; BM	The mRNA expression of **miR-146a** was significantly increased in children with ALL compared with controls (*p* < 0.05).
Liu et al., 2019 [49]	30 childhood T-ALL patients and 30 controls; China	qRT-PCR; whole blood	Significantly higher expression of **miR-663b** in children with T-ALL compared with controls (*p* < 0.01).
Piatopoulou et al., 2018 [50]	125 childhood ALL cases at diagnosis and 64 controls; Greece	qRT-PCR; BM	Levels of **miR-143** and **miR-182** were significantly lower in ALL patients compared to controls (*p* < 0.001). Univariate logistic regression for miR-143 (OR, 0.108; 95% CI: 0.056 to 0.207; *p* < 0.001) and miR-182 (OR, 0.221; 95% CI: 0.126 to 0.386; *p* < 0.001) and ROC curve analysis revealed a strong clinical significance of both miR-143 and miR-182 in differential diagnosis of childhood ALL (AUC, 0.88; 95% CI: 0.831 to 0.928; *p* < 0.001 and AUC, 0.762; 95% CI: 0.694 to 0.829; *p* < 0.001; respectively). Multivariate logistic regression analysis highlighted that lower levels of miR-143 (OR, 0.115; 95% CI: 0.054 to 0.245; *p* < 0.001) can discriminate leukemic from normal BM specimens, independently of patient age and gender.
Swellam et al., 2018 [51]	43 childhood ALL patients at diagnosis and 23 controls; Egypt	qRT-PCR; whole blood	Expression levels of **miR-125b-1** were 32.6-fold higher in ALL cases as compared to controls (median: 66.67 vs. 2.04; *p* < 0.0001), whereas expression of **miR-203** was 30.76-fold higher in controls than in ALL patients (median: 20.34 vs. 0.66; *p* < 0.0001). ROC analysis for miR-203 displayed better sensitivity scores than miR-125b-1: (a) miR-203: AUC, 0.874; 95% CI: 0.769 to 0.942; *p* < 0.0001; cutoff 0.973; 97.7% sensitivity; 87% specificity, (b) miR-125b-1: AUC, 0.858; 95% CI: = 0.715 to 0.847; *p* < 0.0001; cutoff 3.209; 83.7% sensitivity; 100% specificity; (c) miR-125b-1 combined with miR-203: 100% sensitivity; 87% specificity; 93.5% positive predictive value; 100% negative predictive value; 95.5% accuracy. These two miRNA expression levels were negatively correlated (r = −0.302; *p* = 0.014).
Ghodousi et al., 2018 [52]	46 childhood ALL patients at diagnosis and 16 controls; Iran	qRT-PCR; BM	Both **miR-326** and **miR-200c** expression levels were significantly lower in ALL patients than controls (*p* = 0.0002 and 0.035, respectively). ROC curve analysis revealed a high AUC for miR-326 (0.813; 95% CI: 0.671 to 0.954; cutoff, 0.29; sensitivity, 83.3%; and specificity, 70.8%; *p* < 0.001), whereas the AUC for miR-200c was 0.79 (95% CI: 0.649 to 0.932; cutoff, 0.42; sensitivity, 66.7%; and specificity, 71.4%; *p* = 0.004).
Shafik et al., 2018 [53]	70 childhood ALL patients at diagnosis and 7 controls; Egypt	qRT-PCR; BM	Expression of **miR-128** was significantly elevated in ALL patients compared with controls (*p* < 0.001).
Jiang et al., 2018 [54]	92 children with ALL (both at diagnosis and relapsed) and 3 controls; China	qRT-PCR; BM and plasma	Expression of **miR-652**-3p was significantly lower in ALL patients at diagnosis compared with healthy controls (*p* < 0.05). In the same study, a total of 45 miRNAs were documented as significantly downregulated in newly diagnosed patients compared with controls (i.e., 3661.7-fold downregulation of **miR-22** in ALL patients compared to controls; *p* = 00002): miR-22, **miR-624**, miR-29a*, **miR-183**, **miR-190b**, **miR-339**-5p, **miR-501**-3p, **miR-200b**, **miR-195**, mmu-**miR-140**, **miR-487b**, **miR-301**, **miR-200c**, **miR-106b**, **miR-502**-3p, **miR-18a**, **miR-27a**, SNORD48 X2, **miR-142**-5p, **miR-142**-3p, miR-15a*, **miR-196b**, miR-340*, miR-183*, miR-191*, **miR-205**, **miR-370**, **miR-29a**, **miR-532**-3p, **miR-532**, **miR-324**-3p, **miR-20b**, **miR-138**, **miR-26b**, **miR-433**, **miR-550**, **miR-19b**, miR-136*, **miR-1290**, **miR-374**, **miR-1180**, **miR-642**, **miR-10b**, mmu-**miR-93**, **miR-15b**, miR-20a*, **miR-500**, **miR-223**, miR-652, **miR-20a**, **miR-27b**, **miR-19a**, dme-**miR-7**, miR-22*, **miR-30c**, **miR-30b** and **miR-769**-5p.
Zang et al., 2018 [55]	81 childhood ALL patients and 83 controls; China	qRT-PCR; whole blood	Significant downregulation of **miR-9** in T-cells obtained from ALL patients relative to controls (*p* < 0.05). Induced overexpression of miR-9 inhibited ALL development in vitro via its downstream target, neuropilin-1 (NRP1).
Asnafi et al., 2017 [56]	41 children with ALL and 41 controls; Iran	qRT-PCR; BM and whole blood	Expression of **miR-21** and **miR-150** was downregulated in ALL patients compared with controls (unknown significance), whereas miR-451 expression displayed no difference.
Piatopoulou et al., 2017 [57]	125 childhood ALL patients at diagnosis and 64 controls; Greece	qRT-PCR; BM	Expression analysis revealed the significant downregulation of **miR-125b** levels in childhood ALL patients compared to controls (*p* < 0.004). The discriminatory significance of miR-125b for childhood ALL from healthy children was confirmed by univariate logistic regression (OR, 0.477; 95% CI: 0.288 to 0.790; *p* = 0.004) and ROC analysis (AUC, 0.628; 95% CI: 0.548 to 0.707; *p* = 0.004). The discriminatory value of miR-125b seems to be independent of patient age and gender according to the adjusted multivariate logistic regression model (OR, 0.507; 95% CI: 0.305 to 0.842; *p* = 0.009).
Nabhan et al., 2017 [58]	30 childhood ALL patients at diagnosis and 30 controls; Egypt	qRT-PCR; serum	Significant downregulation of **miR-181a** in children with ALL compared with controls (*p* < 0.01). The previous finding were associated with SMAD7 overexpression and TGF-β1 downregulation (increasing proliferation and decreasing apoptosis). ROC curve analysis revealed an AUC of 0.93 (cutoff, 0.97; 86.5% sensitivity; 93.3% specificity; miR-181a positivity rate, 86.7%; *p* < 0.01). Serum levels of SMAD7, TGF-β1 (cutoff values ≥400.1 pg/mL and ≤0.25 ng/mL, respectively) and miR-181a seem to discriminate ALL cases from controls with 100% sensitivity, 93.3% specificity, 96.7% accuracy, 100% negative predictive value and 93.7% positive predictive value.
Yuan et al., 2017 [59]	111 childhood ALL patients and 111 controls with fractures; China	qRT-PCR; plasma	The expression levels of **let-7f**-5p, **miR-5100** and **miR-25**-3p in ALL patients were significantly lower than in controls (*p* < 0.01). After adjusting for confounders, risk for ALL (OR and 95% CI) was calculated to be 0.84 (0.76 to 0.92), 0.81 (0.73 to 0.9) and 0.81 (0.74 to 0.89), respectively (*p* < 0.01 in all instances). ROC analysis and reclassification analysis showed that utilization of all three miRNAs, compared to established risk factors, can improve the AUC and improve diagnosis (0.78; 95% CI: 0.72 to 0.84; *p* = 0.012).
Labib et al., 2017 [60]	75 childhood B-ALL patients at diagnosis and 50 controls; Egypt	qRT-PCR; BM and serum	Significant upregulation of **miR-21** in children with B-ALL compared to controls (9.62 ± 3.23 vs. 2.56 ± 0.83; *p* < 0.001; cutoff, 9.83 for high and low expression). ROC curve analysis revealed an AUC of 0.879 (88.7% sensitivity; 71.8% specificity; cutoff, 3.23) in distinguishing ALL patients from controls.
Tian et al., 2017 [61]	189 childhood B-ALL cases and 189 controls; China	qRT-PCR; N/A	Significant downregulation of **miR-3173** in children with B-ALL compared to controls (*p* < 0.001).
Ramani et al., 2017 [62]	60 childhood B-ALL patients and 17 controls; the Netherlands	qRT-PCR; BM or PBMCs	The analysis revealed a signature of 136 significantly (*p* < 0.05; 45 of them with *p* < 10^−5^) differentially expressed miRNAs distinguishing pediatric patients with ALL from controls. Significant upregulation in ALL: **miR-133b**, **miR-302**, **miR-190**, **miR-520**, **miR-10b**, **miR-515**-5p, **miR-517b**, **miR-501**, **miR-129**, **miR-155**, **miR-217**, **miR-330**, **miR-513**, **miR-585**, **miR-645**, **miR-617**, **miR-15b**, **miR-23a**, **miR-362**, **miR-368**, **miR-425**-5p, **miR-576** and **miR-369**-5p. Significant downregulation in ALL: **miR-193b**, **miR-325**, **miR-514**, **miR-22**, **miR-7g**, **miR-7d**, **miR-302d**, **miR-206**, **miR-494**, **miR-101**, **miR-126**, **miR-100**, **miR-29a**, **miR-299**-3p, **miR-146**, **miR-20a**, **miR-374**, **miR-216**, **miR-532**, **miR-25**, **miR-30c** and **miR-30e**-3p.
Cao et al., 2016 [63]	42 childhood ALL patients and 20 controls; China	qRT-PCR; BM	Significant downregulation of **miR-34b** in ALL patients compared with controls (1.65 ± 0.69 vs. 5.22 ± 1.15; *p* = 0.012).
Lou et al., 2016 [64]	20 childhood B-ALL patients and 20 controls; China	qRT-PCR; BM	Expression of **miR-187**-5p was significantly upregulated in B-ALL samples compared with controls (*p* < 0.001). This miRNA seems to modulate the Wnt/b-catenin signaling pathway via direct targeting of DDK2, thus promoting ALL cell proliferation and inhibiting apoptosis.
Swellam et al., 2016 [65]	85 childhood ALL patients at diagnosis and 25 controls; Egypt	qRT-PCR; PBMCs	Significant upregulation of **miR-100** (median: 61.777 vs. 2.04; range: 0.14–259.39 vs. 0.173–3.029; *p* = 0.007) and **miR-146a** (121.3 vs. 2.05; 14.3–222.55 vs. 0–144; *p* < 0.0001) in ALL patients compared with controls. Significant downregulation of **miR-196a** (0.369 vs. 0.511; 0.001–144 vs. 0.08–0.973; *p* = 0.028) in ALL samples compared with controls. ROC analysis revealed a significant AUC for miRNA-146a (1; 95% CI: 0.956 to 1; SE 0; 100% sensitivity; 100% specificity; cutoff, 3.727; *p* < 0.0001) and miR-100 (0.87; 95% CI: 0.779 to 0.934; SE 0.038; 82.76% sensitivity; 100% specificity; cutoff, 3.029; *p* = 0.0001) in discriminating ALL patients from controls.
de Oliveira et al., 2015 [66]	128 childhood ALL patients at diagnosis and 11 controls; Brazil	qRT-PCR; BM	Significantly higher **miR-708**-5p expression in ALL samples compared with controls (*p* < 0.05). High expression was documented specifically for pre-B ALL (*p* < 0.01).
Wu et al., 2015 [67]	40 infants with *KMT2A*-rearranged ALL (26 *KMT2A*-*AFF1*, 4 *KMT2A*-*MLLT3*, 5 *KMT2A*-*MLLT1* and 5 *KMT2A*-germline) at diagnosis and 8 controls; Japan	qRT-PCR; BM or whole blood	In infant ALL cells with *KMT2A* fusion, **let-7b** was significantly downregulated compared with controls according to DNA hypermethylation of its gene-promoter region.
Nemes et al., 2015 [68]	24 childhood ALL patients and no controls; Hungary	qRT-PCR; bone marrow and whole blood	Significant downregulation of **miR-21**, **miR-24** and **miR-29b** in T-ALL patients compared to controls (*p* < 0.05). Significant upregulation of **miR-155** in B-ALL patients compared to controls (*p* < 0.05). Significant upregulation of **miR-128b** in both B- and T-ALL children compared with controls (*p* < 0.05). Expression profiles of miRNAs were not significantly different between peripheral blood and bone marrow samples derived from the same patient.
Luna-Aguirre et al., 2015 [69]	19 childhood B-ALL patients at diagnosis and 7 adult controls; Mexico	qRT-PCR; plasma	A total of 40 significantly overexpressed miRNAs in B-ALL patients compared to controls: **miR-511**, miR-34a*, **miR-565**, **miR-34a**, miR-10b*, **miR-630**, **miR-610**, **miR-181a**, **miR-181c**, **miR-222**, miR-138-1*, **miR-363**, miR-144*, **miR-451**, **miR-99a**, **miR-155**, **miR-886**-3p, miR-223*, **miR-422a**, **miR-146a**, **miR-192**, **miR-190b**, **miR-95**, **miR-140**-3p, **miR-660**, miR-886-5p, **miR-25**, **miR-320**, **miR-30e**, **miR-16**, **miR-19b**, **miR-500**, **miR-29a**, **miR-502**-3p, **miR-195**, **miR-20b**, **miR-579**, **miR-7**, **miR-19a** and **miR-768**-3p. A total of 37 miRNAs significantly downregulated in B-ALL patients compared with controls: **miR-199a**-3p, miR-340*, **miR-151**-3p, **miR-335**, **miR-99b**, miR-425*, **miR-224**, **miR-221**, **miR-744**, **miR-15b**, **miR-223**, **miR-26a**, miR-454*, **miR-452**, **miR-491**-5p, **miR-340**, **miR-196b**, **miR-301a**, **miR-324**-5p, miR-126*, **miR-152**, **miR-330**-3p, **miR-652**, **miR-374b**, **miR-148b**, **miR-671**-3p, **miR-18a**, **let-7d**, **miR-339**-3p, **miR-126**, **miR-30b**, miR-148b*, **miR-27a**, **miR-30c**, **miR-374a**, **miR-331**-3p and **miR-28**-5p. Diagnostic capacity for miR-511 (overexpressed in B-ALL; range, 3.32 to 12.98; cutoff, 9.458; AUC, 1; 100% sensitivity, specificity, PPV and NPV), miR-34a (overexpressed; range, 0.73 to 7.55; cutoff, 7.179; AUC, 0.98; 92% sensitivity; 100% specificity and PPV; 70% NPV), miR-222 (overexpressed; range, −1.5 to 3.12; cutoff, −0.1325; AUC, 0.91; 79% sensitivity; 100% specificity and PPV; 54% NPV), miR-26a (underexpressed; range, −1.63 to −9.17; cutoff, 2.073; AUC, 0.91; 79% sensitivity; 100% specificity and PPV; 47% NPV), miR-221 (underexpressed; range, 1.1 to −8.21; cutoff, −0.1861; AUC, 0.92; 83% sensitivity; 100% specificity and PPV; 54% NPV) and miR-223 (underexpressed; range, 0.31 to −8.52; cutoff, −4.309; AUC, 0.93; 89% sensitivity; 100% specificity and PPV; 64% NPV) showed that miR-511 could be used as a circulating biomarker for B-ALL detection. Moreover, miR-199a-3p (most underexpressed compared to controls; RQ, -13.48; *p* < 0.001), along with miR-511 (most overexpressed; RQ, 159.55; *p* = 0.002), might be associated with the pathogenesis of B-ALL.
Organista-Nava et al., 2015 [70]	111 childhood ALL patients and 100 controls; Mexico	qRT-PCR; BM and/or whole blood	Significantly lower **miR-24** expression in ALL patients compared with controls (median, 0.84; *p* = 0.002).
Oliveira et al., 2015 [71]	37 childhood T-ALL patients and normal T-cells; Brazil	qRT-PCR; BM or whole blood	Significantly lower **miR-29a** expression in T-ALL patients compared with control T-cells.
Duyu et al., 2014 [28]	43 childhood ALL patients at diagnosis and 14 controls; Turkey	qRT-PCR and microarray; BM and whole blood	A total of 13 miRNAs (miR-548i, miR-708, miR-181b, miR-449a, miR-146a, miR-155, miR-181a*, miR-3121, miR-181a, miR-128, miR-1323, miR-195 and miR-587) showed upregulation, and 2 miRNAs (miR-640 and miR-145) showed downregulation in the microarray study. Confirmation analysis by qRT-PCR demonstrated only five upregulated miRNAs: **miR-128**, **miR-146a**, **miR-155**, **miR-181a** and **miR-195** (*p* < 0.05). Microarrays revealed significant upregulation of miR-548i, miR-3140, miR-181b, miR-3115, miR-548d-5p, miR-449a, miR-181c, miR-181a, miR-181a*, let-7b*, miR-1827, miR-92a-1*, miR-299-3p, miR-155, miR-181a-2*, miR-1323, miR-587, miR-7-1*, miR-28-3p, miR-130b, miR-27b and miR-361-5p and significant downregulation of miR-633, miR-326, miR-501-3p, miR-802, miR-4260, miR-3130-5p, miR-145, miR-186, miR-593*, miR-574-3p, miR-4262, miR-640 and miR-606 in T-ALL patients compared to controls. Significant upregulation of miR-708, miR-181b, miR-369-3p, miR-146a, miR-155, miR-195 and miR-128 and significant downregulation of miR-143 and miR-145 in children with B-ALL compared with healthy controls. Intriguingly, miRNAs from peripheral blood samples were not correlated with BM samples.
Malik et al., 2014 [72]	30 childhood B-ALL and 20 T-ALL patients and 50 controls; India	qRT-PCR; PBMCs	Significantly higher expression of **miR-2909** in both B- and T-ALL samples compared with controls (*p* < 0.01).
Gimenes-Teixeira et al., 2013 [73]	48 childhood T-ALL patients and 10 controls; Brazil	qRT-PCR; BM, PBMCs and thymic samples	Significantly higher **miR-221** and **miR-374** expression in T-ALL samples compared with controls (*p* < 0.05).
Xue Li et al., 2013 [74]	34 childhood common-ALL patients and 5 controls; China	Microarray; BM	Significantly upregulated expression of **miR-708** (16.886 ± 16.854 vs. 1.872 ± 0.339; *p* < 0.01), **miR-181b** (5.710 ± 4.652 vs. 1.276 ± 0.531; *p* = 0.006) and **miR-210** (9.789 ± 1.178 vs. 1.005 ± 0.08; *p* < 0.01) in children with common ALL compared with controls. Significant downregulation of **miR-345** (0.675 ± 0.086 vs. 1.204 ± 0.143; *p* = 0.007) and **miR-27a** (0.523 ± 0.085 vs. 1.123 ± 0.066; *p* = 0.004) in common ALL compared with controls.
Li et al., 2013 [75]	111 childhood ALL patients and 10 controls; China	qRT-PCR; BM	The expression levels of **miR-100** and **miR-99a** were significantly downregulated in ALL patients compared with controls (*p* = 0.001 and *p* = 0.0086, respectively).
de Oliveira et al., 2012 [76]	128 childhood ALL patients at diagnosis and 11 controls; Brazil	qRT-PCR; BM	Significantly lower expression of **miR-100** (median, 0.21; range, 0.002 to 10.76; vs. 1.04 (0.34 to 3.9); *p* < 0.01)), **miR-196b** (0.03 (0.001 to 65.68) vs. 1.07 (0.29 to 3.7); *p* < 0.01) and **let-7e** (0.59 (0.04 to 5.15) vs. 1.16 (0.54 to 3.25); *p* < 0.01) and significantly higher expression of **miR-128a** (4.32 (0.16 to 167.91) vs. 1.02 (0.57 to 2.23); *p* < 0.01) and **miR-181b** (5.06 (0.07 to 45.09) vs. 1.41 (0.33 to 3.4); *p* < 0.01) in ALL samples compared to controls. The expression of miR-92a and miR-125a-5p was similar for both groups.
Borze et al., 2011 [77]	9 childhood ALL patients and 8 controls of N/A age; Finland	Microarray; BM	Significant upregulation of **miR-142**-3p (*p* = 0.001), **miR-146a** (*p* = 0.004), **miR-222** (*p* = 0.012), miR-142-5p (*p* = 0.022), miR-150* (*p* = 0.032), **miR-144** (*p* = 0.041) and **miR-155** (*p* = 0.043) in ALL samples compared with controls. Significant downregulation of **miR-768**-5p (*p* < 0.001), **miR-125b** (*p* = 0.006), **miR-223** (*p* = 0.007), **miR-22** (*p* = 0.012), **miR-27a** (*p* = 0.027) and **miR-15b** (*p* = 0.032) in ALL patients compared with healthy donors. Similar expression levels of miRNAs between BM core biopsies (under decalcification and fixation with formalin) and BM aspirates.
Bhatia et al., 2011 [78]	10 childhood T-ALL patients at diagnosis and control T-cells; India	Semi-qRT-PCR; PBMCs	Significantly lower expression levels of **miR-196b** and **miR-148a** and significantly higher expression levels of **miR-30b** and **miR-151** in children with T-ALL compared with normal T-cells (*p* < 0.05). Consequently, miR-196b was unable to downregulate *MYC* protooncogene expression in T-ALL patients as a result of mutations in the 3′-untranslated region (3′-UTR) of the target.
Mavrakis et al., 2011 [79]	50 childhood T-ALL patients at diagnosis and normal T-cell populations from pediatric thymuses; Belgium and France	qRT-PCR; BM and thymic T-cells	Significantly higher **miR-223**, **miR-19b**, **miR-20a**, **miR-92**, **miR-142**-3p, **miR-150**, **miR-93**, **miR-26a**, **miR-16** and **miR-342** expression in T-ALL samples compared with controls (*p* < 0.05).
Stumpel et al., 2011 [80]	5 t(4;11)-positive ALL infants at diagnosis and 7 controls; the Netherlands	qRT-PCR; BM	Significant downregulation of **miR-10a**, **miR-101**, **miR-148a**, **miR-152**, **miR-200a**, **miR-200b**, **miR-424**, **miR-429**, **miR-432**, **miR-486** and **miR-503** in *KMT2A*-rearranged ALL samples compared with controls (via hypermethylation).
Schotte et al., 2011 [81]	70 childhood ALL patients at diagnosis and 10 controls; the Netherlands	RNA-seq and qRT-PCR; BM and PBMCs	Significant downregulation of **miR-3136** and **miR-4474** in ALL patients compared to controls.
Bhatia et al., 2010 [82]	10 ALL patients of N/A age and normal B-cells; India	RT-PCR; PBMCs	Significant downregulation of **miR-151**, **miR-196b** and **miR-148a** in patient samples compared with B-cells obtained from healthy volunteers (*p* < 0.05). Among them, only miR-196b was found to be downregulated in the EB-3 cell line (potential tumor-suppressive function), whereas it seemed to downregulate the highly expressed *MYC* and, in turn, to decrease the expression of *MYC* effector genes (*TERT*, *BCL2* and *AATF*).
Schotte et al., 2009 [83]	92 childhood ALL patients at diagnosis (20 *MLL*-rearranged, 57 B-ALL and 15 T-ALL) and normal CD34^+^ progenitor cells from 2 children with brain tumors; the Netherlands	qRT-PCR; BM or whole blood	Significant upregulation of **miR-128a**, **miR-142**-3p and -5p, as well as **miR-150**, **miR-151**-5p (not in *KMT2A*-rearranged cases), **miR-181a**, **miR-181b**, **miR-181c**, **miR-193a**, **miR-30e**-5p, **miR-34b**, **miR-365**, **miR-582** and **miR-708** (*p* ≤ 0.001) in ALL patients compared with controls. Significant downregulation of **miR-100**, **miR-125b**, **miR-99a** and **let-7e** in children with ALL compared with controls (*p* < 0.001). Conversely, **miR-196b** was found to be significantly downregulated in B-other cases (*p* < 0.001) and significantly upregulated in *KMT2A*-rearranged cases (*p* = 0.021) compared to controls.
Zhang, Yang, et al., 2009 [84]	24 childhood ALL patients at diagnosis and 3 controls; China	qRT-PCR; BM	Significantly higher expression levels of **miR-9**, miR-9*, **miR-181a**, **miR-128**, **miR-181b**, **miR-130b** and **miR-363** in children with ALL compared with controls. Significantly lower expression of **miR-7e**, **miR-30a**, **miR-199b**-3p, **miR-126**, **miR-143**, **miR-223** and **miR-582**-5p in ALL samples compared to controls. With respect to novel miRNAs **miR-1943**, **miR-1841**, **miR-1931**, **miR-1987**, **miR-1890** and **miR-1902** were upregulated, and **miR-1859**, miR-1859*, **miR-1947**, **miR-1971**, **miR-1866**, **miR-1986**, **miR-1843**, **miR-1852**, miR-1852*, **miR-1842**, **miR-1834**, miR-1971* and **miR-1893** were down regulated in ALL specimens compared with controls (*p* < 0.001).
Ju et al., 2009 [85]	40 childhood pre-B-ALL patients at diagnosis and 6 controls; China	Microarray and qRT-PCR; BM	Significantly higher expression levels of **miR-339** (*p* < 0.001), **miR-222** (*p* = 0.0061) and **miR-142**-3p (*p* < 0.001) and significantly lower expression of **miR-451** and miR-373* (*p* < 0.001) in ALL samples compared with controls. Upregulation of miR-361, miR-487b and miR-519e and downregulation of miR-296, miR-485-3p and miR-483 in ALL specimens compared with controls in microarrays, although not been confirmed by qRT-PCR.
Zhang, Luo, et al., 2009 [86]	18 childhood ALL patients and 7 controls (validation cohort: 31 ALL cases and 5 controls); China	Microarray and qRT-PCR; BM	Significantly higher expression of **miR-146a**, **miR-34a**, **miR-210**, **miR-213**, **miR-181c**, **miR-126**, **miR-181a**, **miR-181d**, **miR-130a**, **miR-195**, **miR-181b**, **miR-130b**, **miR-155**, **miR-17**-3p, **miR-128b**, **miR-128a**, **miR-18b**, **miR-28**, **miR-331**, **miR-505** and **miR-363** and significantly lower expression of **miR-582**, **miR-142**-5p, **miR-145**, **miR-143**, **miR-338**, **miR-148a**, **miR-424**, **miR-24**, **miR-29a**, **miR-199b** and **miR-199a** in children with ALL compared with controls.

AATF = apoptosis-antagonizing transcription factor; BCL2 = B-cell lymphoma 2 apoptosis regulator; BCP-ALL = B-cell precursor ALL; BM = bone marrow samples; CBL = Casitas B lineage lymphoma proto-oncogene; CDK6 = cyclin-dependent kinase 6; HRM = high-resolution melting analysis; IRF4 = interferon regulatory factor 4; MAPK = mitogen-activated protein kinase; MMP = matrix metallopeptidase; MYC = MYC proto-oncogene, BHLH transcription factor; N/A = not available; PBMCs = peripheral blood mononuclear cells; PTK2 = protein tyrosine kinase 2; ROC curve = receiver operating characteristic curve; SMAD7 = mothers against decapentaplegic homolog or SMAD family member 7; T-ARMS-PCR = tetra-primer amplification refractory mutation system PCR; TERT = telomerase reverse transcriptase; TGF-β1 = transforming growth factor beta 1; TLDA = TaqMan low-density array. MicroRNAs in bold indicate statistically significant differential expression.

## Supplementary Materials

The following supporting information can be downloaded at: https://www.mdpi.com/article/10.3390/cancers14163976/s1, Supplementary Table S1: Upregulated and downregulated miRNAs in childhood ALL subtypes; Supplementary Table S2: Results of reanalysis of small RNA-seq data of pediatric T-ALL cases compared with controls. References [216,217,218,219,220,221,222,223] are cited in Supplementary Materials.
