# Supplementary material for: MicroRNAs and the Diagnosis of Childhood Acute Lymphoblastic Leukemia: Systematic Review, Meta-Analysis and Re-Analysis with Novel Small RNA-Seq Tools
Source: Cancers (Basel). 2022 Aug 17;14(16):3976. doi: 10.3390/cancers14163976 (PMC9406077; doi:10.3390/cancers14163976)
Supplement: Supplementary file 1 [file cancers-14-03976-s001.zip › Supplementary Table S1.pdf]

Supplementary Table S1. Upregulated and downregulated miRNAs in childhood ALL subtypes.

Subtype	Upregulation	Downregulation
T-ALL vs controls	let-7b* [1], miR-7-1* [1], miR-16 [2, 3], miR-18b [4], miR-19b [2, 5], miR-20a [2], miR-20b [4], miR-26a [2], miR-27a [5], miR-27b [1], miR-28 [1], miR-29b [5], miR-30b [6], miR-30e [2], miR-92 [2], miR-92a-1* [1], miR-93 [2], miR-128 [4], miR-128b [7], miR-130a [4], miR-130b [1, 4], miR-142 [2], miR-144 [5], miR-146a [2, 8], miR-150 [2], miR-151 [6], miR-153 [4, 5], miR-155 [1], miR-181a [1, 4], miR-181a* [1], miR-181a-2 [4], miR-181a-2* [1], miR-181b [1, 4], miR-181c [1], miR-210 [4], miR-221 [8, 9], miR-223 [2], miR-299 [1], miR-331 [4], miR-342 [2], miR-361 [1], miR-363 [4], miR-374 [9], miR-376a [2], miR-449a [1], miR-466 [4], miR-486 [5], miR-548a [4], miR-548d [1], miR-548i [1], miR-582 [5], miR-587 [1], miR-625 [4], miR-662 [2], miR-663b [10], miR-1277 [5], miR-1307 [5], miR-1323 [1], miR-1827 [1], miR-	miR-7 [2], miR-10a [4], miR-10b [4], miR-21 [7], miR-23a [4], miR-24 [7], miR-24-2 [4], miR-27a [4], miR-29a [2, 12], miR-29b [7], miR-30a [4], miR-100 [13], miR-143 [4], miR-145 [4], miR-148a [6], miR-151a [4], miR-195 [2], miR-196b [6], miR-199b [4], miR-210 [13], miR-223 [4], miR-296 [2], miR-326 [1], miR-335 [14], miR-338 [4], miR-451 [15], miR-501 [1], miR-504 [4], miR-550a [4], miR-574 [4], miR-582 [4], miR-606 [1], miR-618 [4], miR-633 [1], miR-640 [1], miR-802 [1], miR-941 [4], miR-1275 [4], miR-2115 [4], miR-3150b [4], miR-3154 [4], miR-3690 [4], miR-3909 [4], miR-4260 [1], miR-4494 [4], miR-4695 [4], miR-4745 [4], miR-6823 [4], miR-6865 [4], miR-7849 [4]

	2909 [11], miR-3115 [1], miR-3140 [1], miR-3609 [4], miR-4421 [4], miR-4437 [4], miR-4687 [4], miR-6500 [4]	
B-ALL vs controls	miR-7 [16], miR-10b [17], miR-10b* [16], miR-15b [17], miR-16 [16, 18], miR-19a [16], miR-19b [16], miR-20b [16], miR-21 [18, 19], miR-23a [17], miR-25 [16], miR-29a [16], miR-30d [18], miR-30e [16], miR-34a [16, 18], miR-34a* [16], miR-92a [18], miR-93 [18], miR-95 [16], miR-99a [16], miR-100 [13, 20], miR-125b [18], miR-128 [1, 18], miR-128b [7], miR-129 [17], miR-133b [17], miR-138-1* [16], miR-140 [16], miR-142 [21], miR-144* [16], miR-146a [1, 16, 18], miR-155 [1, 7, 16–18], miR-181a [16, 18], miR-181b [1, 18, 22], miR-181c [16, 18], miR-187 [23], miR-190 [17], miR-190b [16], miR-192 [16], miR-195 [1, 16], miR-210 [13, 22], miR-217 [17], miR-222 [16, 18, 21], miR-223* [16], miR-302 [17], miR-320a [16, 18], miR-330 [17], miR-339 [21], miR-361 [18], miR-362 [17], miR-363 [16], miR-	let-7d [16], miR-7d [17], miR-7g [17], miR-15b [16], miR-18a [16], miR-20a [17], miR-22 [17], miR-25 [17], miR-26a [16], miR-27a [16, 22], miR-28 [16], miR-29a [17], miR-30b [16], miR-30c [16, 17], miR-30e [17], miR-99b [16], miR-100 [17], miR-101 [17], miR-126 [17], miR-126 [16], miR-126* [16], miR-143 [1], miR-145 [1], miR-146 [17], miR-148b [16], miR-148b* [16], miR-151 [16], miR-152 [16], miR-193b [17], miR-196b [16, 25], miR-199a [16], miR-206 [17], miR-216 [17], miR-221 [16], miR-223 [16, 18], miR-224 [16], miR-299 [17], miR-301a [16], miR-302d [17], miR-324 [16], miR-325 [17], miR-326 [26], miR-330 [16], miR-331 [16], miR-335 [16], miR-339 [16], miR-340 [16], miR-340* [16], miR-345 [22], miR-373* [21], miR-374a [16–18], miR-374b [16], miR-425* [16], miR-451 [21], miR-452 [16], miR-454* [16], miR-491 [16], miR-494 [17, 18], miR-514

	368 [17], miR-369 [1, 17], miR-422a [16], miR-425 [17], miR-451 [16], miR-500 [16], miR-501 [17], miR-502 [16], miR-511 [16], miR-513 [17], miR-515 [17], miR-517b [17], miR-520 [17], miR-532 [18], miR-565 [16], miR-576 [17], miR-579 [16], miR-585 [17], miR-610 [16], miR-617 [17], miR-630 [16], miR-645 [17], miR-660 [16, 18], miR-708 [1, 22, 24], miR-768 [16], miR-886 [16], miR-2909 [11]	[17], miR-532 [17], miR-652 [16], miR-671 [16], miR-744 [16], miR-3173 [27]
KMT2A-r vs controls	miR-30e [25], miR-34b [25], miR-128a [25], miR-142 [25], miR-150 [25], miR-181a [25], miR-181b [25], miR-181c [25], miR-193a [25], miR-196b [25], miR-223 [2], miR-365 [25], miR-376a [2], miR-506 [28], miR-582 [25], miR-662 [2], miR-708 [25], miR-922 [28]	let-7b [29, 30], let-7e [25], miR-7 [2], miR-10a [31], miR-29a [2], miR-99a [25, 32], miR-100 [25, 32], miR-101 [31], miR-125b [25], miR-148a [31], miR-152 [31], miR-195 [2], miR-200a [31], miR-200b [31], miR-424 [31], miR-429 [31], miR-432 [31], miR-486 [31], miR-503 [31]
Hyperdiploid vs controls	miR-128 [18], miR-146a [18], miR-181b [18], miR-222 [18], miR-365 [33], miR-532 [18]	
t(12;21) vs controls	miR-128 [18], miR-146a [18], miR-181b [18], miR-222 [18], miR-532 [18]	

T-ALL vs B-ALL	miR-29c [34], miR-100 [35], miR-190 [36], miR-196a [35], miR-196b [37–39], miR-322 [37], miR-342 [36], miR-424 [34], miR-450a [34], miR-450b [34], miR-542 [34, 36, 40], miR-629 [34], miR-1246 [37], miR-1273 [37], miR-1290 [37], miR-1915 [37], miR-3136 [41]	miR-21 [42], miR-34a [37], miR-99a [32], miR-100 [32], miR-125b [43], miR-126 [37], miR-132 [36], miR-150 [37], miR-151a [34, 36, 37], miR-151b [34], miR-191 [36], miR-195 [34], miR-222 [37], miR-222* [36], miR-371 [34], miR-425 [34, 36], miR-451 [37], miR-455 [34], miR-497 [34, 37], miR-574 [34], miR-708 [24, 34, 36, 37, 40, 41, 44], miR-1266 [34], miR-3150b [41]
B-other vs controls	miR-30e [25], miR-34b [25], miR-128a [25], miR-142 [25], miR-150 [25], miR-151 [25], miR-181a [25], miR-181b [25], miR-181c [25], miR-193a [25], miR-365 [25], miR-582 [25], miR-708 [25]	let-7e [25], miR-99a [25], miR-100 [25], miR-125b [25], miR-196b [25]
Biphenotypic vs ALL	miR-146a [35]	
ETV6-RUNX1 vs BCP-ALL or pre-B-ALL	let-7c [45, 46], <u>miR-99a</u> [36, 45, 46], <u>miR-100</u> [36, 45], <u>miR-125b</u> [36, 45], miR-125b-2 [46], miR-126 [36, 45], miR-126* [36], miR-151 [37], <u>miR-218</u> [45], miR-320b-1 [47], miR-345	let-7a [48], let-7b [48], miR-19a [48], miR-30e [48], miR-92 [48], miR-130b [48], miR-155 [48], miR-181a-1 [48], miR-181c [48], miR-181d [48], miR-195 [48], miR-200c [45, 47], miR-213 [36], miR-221 [36, 45, 48], miR-222 [45, 48], miR-320a [47, 49],

	[45], miR-383 [36], miR-629 [36], miR-922 [47], miR-4747 [47]	miR-342 [48], miR-361 [36], miR-423 [48], miR-425 [48], miR-494 [49], miR-660 [48], miR-1976 [47]
ETV6-RUNX1 vs ALL	miR-100 [38], miR-335 [50], miR-3117 [41], miR-3136 [41], miR-3150b [41], miR-3154 [41], miR-5195 [41]	
ETV6 del vs ALL	miR-708 [40]	
PAR1 del vs ALL	miR-24 [40], miR-542 [40]	
PAX5 del vs ALL		miR-24 [40], miR-31 [40], miR-128 [40], miR-708 [40]
miR-31 del vs ALL		miR-542 [40], miR-24 [40]
CDKN2A/B del vs ALL		miR-542 [40]
IKZF1 del vs ALL		miR-128 [40]

BCR-ABL1 vs BCP-ALL	miR-17 [51], miR-18 [51], miR-19a [51], miR-19b [51], miR-20a [51], miR-29a [45], miR-146a [45]	miR-32 [45], miR-93 [36, 45], miR-103 [36, 45], miR-106b [45], miR-148b [36], miR-186 [45], miR-210 [36], miR-301 [36], miR-324 [36, 45], miR-331 [36], miR-345 [36, 45], miR-451 [45], miR-484 [36], miR-1226 [36]
BCR-ABL1 vs ALL		miR-708 [44]
BCR-ABL1-like vs BCP-ALL	miR-101 [45]	miR-103 [45], miR-130a [45], miR-152 [45], miR-190 [45], miR-191 [45], miR-197 [45], miR-324 [45], miR-345 [45], miR-545 [45]
BCR-ABL1 vs controls	let-7b [52], miR-17 [51], miR-18 [51], miR-19a [51], miR-19b [51], miR-20a [51], miR-92 [51]	miR-99a [32], miR-100 [32]
ERG-related vs BCP-ALL	let-7c [53], miR-92a [53], miR-99a [53], miR-100 [53], miR-125a [53], miR-125b [53], miR-126 [53], miR-125b-2* [53], miR-181a [53], miR-181d [53], miR-491 [53], miR-1275 [53]	miR-15b [53], miR-24 [53], miR-27a [53], miR-132* [53], miR-155 [53], miR-625 [53]
HOXA-r vs T-ALL	miR-9 [5], miR-10a [5], miR-182 [5], miR-183 [5], miR-196b [5], miR-200a [5], miR-200b [5], miR-429 [5], miR-1468 [5]	

TAL-r vs T-ALL	miR-15b [5], miR-16 [5], miR-16-2 [5], miR-29c [5], miR-130b [5], miR-182 [5], miR-450b [5], miR-454 [5], miR-3615 [5]	
TLX1/3 vs T-ALL	let-7e [5], miR-17 [5], miR-20a [5], miR-92a [5], miR-92b [5], miR-99a [54], miR-99b [5], miR-107 [5], miR-125a [5], miR-125b-2 [54], miR-671 [5]	
TLX1 vs controls	miR-223 [2], miR-376a [2], miR-574 [2], miR-662 [2]	miR-7 [2], miR-29a [2], miR-95 [2], miR-181d [2], miR-195 [2], miR-213 [2], miR-296 [2], miR-345 [2], miR-422a [2]
TLX3 vs controls	miR-30e [2], miR-223 [2], miR-376a [2], miR-662 [2]	miR-7 [2], miR-29a [2], miR-148a [2], miR-195 [2], miR-296 [2]
Immature T- vs T-ALL	miR-21 [5], miR-30b [5], miR-101 [5], miR-126 [5], miR-146a [5], miR-181a [5], miR-191 [5], miR-221 [5], miR-222 [5], miR-425 [5]	
SIL-TAL1 (<T) vs ALL	miR-146a [55]	

SIL-TAL1 (<T) vs controls	miR-182 [2], miR-223 [2], miR-376a [2], miR-662 [2]	miR-7 [2], miR-29a [2], miR-99a [2], miR-195 [2], miR-196a [2], miR-196b [2], miR-296 [2]
CALM-AF10 (<T) vs controls	miR-223 [2], miR-376a [2], miR-662 [2] + miR-196b [39] vs ALL	miR-7 [2], miR-29a [2], miR-195 [2], miR-627 [2]
Inv7 (<T) vs controls	miR-146a [2], miR-223 [2], miR-376a [2], miR-662 [2] + miR-196b [39] in inv(7)(p15q35) vs ALL	miR-7 [2], miR-29a [2], miR-99a [2], miR-195 [2], miR-296 [2], miR-422a [2], miR-532 [2]
T-subtypes vs controls+	miR-182 X4, miR-196b X3, miR-223 X5, miR-376a X5, miR-146a X3, miR-662 X5	miR-7 X5, miR-29a X5, miR-195 X5, miR-296 X4, miR-422a X2, miR-99a X2
SET-NUP214 vs ALL	miR-196b [39]	
14q32 LOH vs ALL		miR-127 [56], miR-382 [56], miR-412 [56], miR-433 [56]
KMT2A-r early relapse vs remission	miR-99a [57], miR-103b [57], miR-548 [57], miR-1973 [57], miR-4260 [57], miR-4436b [57], miR-4498 [57], miR-4507	

	[57], miR-4699 [57], miR-6805 [57], miR-7113 [57], miR-7161 [57]	
Hyperdiploid vs pre-B-ALL	miR-5196 [58]	
Hyperdiploid vs BCP-ALL	miR-98 [36, 45], miR-195 [45], miR-222 [36, 45], miR-222* [36], miR-223 [36, 45], miR-324 [45], miR-342 [45], miR-345 [45], miR-361 [36], miR-374a [36, 45], miR-501 [36], <u>miR-511</u> [36, 45], miR-532 [36], miR-660 [36, 45]	
Hyperdiploid vs ALL	miR-361 [41], miR-3154 [41]	miR-100 [38]
KMT2A-r vs B-ALL	miR-574 [59], miR-6735 [59]	
KMT2A-r vs BCP-ALL	miR-24 [45], miR-133a [36], miR-186 [45], miR-196a [36], <u>miR-196b</u> [25, 39, 45], miR-425 [45], miR-484 [45]	let-7b [30, 36, 45], let-7c [36, 45], let-7e [25], miR-20b [36, 45], miR-30e [25], miR-34b [25], miR-151 [25], miR-192 [36, 45], miR-193 [25], miR-194 [36, 45], miR-372 [36], miR-497 [36], miR-582 [25], miR-708 [25, 36]

KMT2A-r vs ALL	miR-128a [38], miR-181b [38], miR-196b [41], miR-3151 [41], miR-3154 [41]	miR-708 [41], miR-3150b [41] + miR-128b & miR-221 KMT2A-AFF1 [60]
TCF3-PBX1 vs B-ALL	miR-574 [59], miR-6735 [59]	
TCF3-r vs BCP-ALL	miR-191 [45], miR-425 [45]	miR-24 [33, 36, 45], miR-26a [45], miR-29a [36, 45], miR-30d [36], miR-126 [33, 36, 45], miR-126* [36], miR-146a [36, 45], miR-193a [36], miR-223 [45], miR-365 [33, 36, 45], miR-511 [36, 45], miR-545 [36]
TCF3-PBX1 vs ALL	miR-3136 [41], miR-3150b [41], miR-3154 [41]	
BCP-ALL vs B-ALL	miR-574 [59], miR-6735 [59]	
Infant vs childhood T-ALL	miR-18a [61], miR-29c [61], miR-30c [61], miR-125b-2 [61], miR-128-1 [61], miR-128-2 [61], miR-148b [61], miR-183 [61], miR-185 [61], miR-190a [61], miR-200c [61], miR-210 [61], miR-223 [61], miR-324 [61], miR-331 [61], miR-421	let-7b [61], let-7f [61], miR-31 [61], miR-196b [61], miR-205 [61], miR-4485 [61], miR-6503 [61]

	[61], miR-502 [61], miR-561 [61], miR-652 [61], miR-664b [61], miR-671 [61], miR-766 [61], miR-874 [61], miR-1180 [61], miR-1226 [61], miR-1249 [61], miR-1276 [61], miR-1301 [61], miR-1306 [61], miR-3143 [61], miR-3186 [61], miR-3620 [61], miR-3661 [61], miR-3909 [61], miR-3922 [61], miR-4687 [61], miR-5010 [61], miR-5581 [61], miR-5683 [61], miR-6802 [61], miR-6803 [61], miR-6806 [61], miR-6855 [61], miR-6769b [61], miR-6894 [61], miR-7706 [61]	

[18] = BCP-ALL

[22] = common-ALL

[25] = B-other

[21] = pre-B-ALL

[57] = 3 KMT2A-ENL & 3 KMT2A-AF4

[58] = SNP G>A rs10406069 in miR-5196 associated with higher levels and hyperdiploid subtype → AG significantly lower SMC1A expression ($p<0.01$) than GG, a gene involved in sister chromatin cohesion and lower ARHGEF3 expression, a guanine

nucleotide exchange factor for Rho GTPase. We also have to take into account that miR-5196 is hosted in the CD22 gene, where rs10406069 produces a missense mutation (p.Gly745Asp) in exon 12. Although the association could be due to its impact on the host gene, the effect of this SNP in the protein is predicted to be benign.

[13] = common-ALL not just B-ALL

[5] = includes adult cases too

[54] = TLX3-related vs T-ALL

[36] = has data comparing ALL subgroups with normal cells https://www.haematologica.org/article/view/5966

[62] = pro-B-ALL highest DICER expression and lowest AGO2 expression vs controls (pre-B-ALL significant difference -but not in that extent)

ERG-related cases = remarkably favorable outcome despite a high incidence of inauspicious IKZF1 aberrations

References

1. Duyu, M.; Durmaz, B.; Gunduz, C.; Vergin, C.; Yilmaz Karapinar, D.; Aksoylar, S.; Kavakli, K.; Cetingul, N.; Irken, G.; Yaman, Y.; et al. Prospective evaluation of whole genome microrna expression profiling in childhood acute lymphoblastic leukemia. Biomed Res. Int. 2014, 2014. https://doi.org/10.1155/2014/967585.
2. Mavrakis, K.J.; Van Der Meulen, J.; Wolfe, A.L.; Liu, X.; Mets, E.; Taghon, T.; Khan, A.A.; Setti, M.; Rondou, P.; Vandenberghe, P.; et al. A cooperative microRNA-tumor suppressor gene network in acute T-cell lymphoblastic leukemia (T-ALL). Nat. Genet. 2011, 43, 673–678.

3. Tong, L.G.; Wu, W.Z.; Zhang, Y.P.; Zhou, Z.G.; Chen, Y.F.; Huang, W.J.; Xu, H.; Su, Q.Q. [Expression of miR-16 in patients with T lymphoblastic lymphoma/acute lymphoblastic leukemia]. Zhongguo Shi Yan Xue Ye Xue Za Zhi 2014, 22, 99–103. https://doi.org/10.7534/j.issn.1009-2137.2014.01.020.
4. Dawidowska, M.; Jaksik, R.; Drobna, M.; Szarzyńska-Zawadzka, B.; Kosmalska, M.; Sędek, Ł.; Machowska, L.; Lalik, A.; Lejman, M.; Ussowicz, M.; et al. Comprehensive Investigation of miRNome Identifies Novel Candidate miRNA-mRNA Interactions Implicated in T-Cell Acute Lymphoblastic Leukemia. Neoplasia 2019, 21, 294–310, https://doi.org/10.1016/j.neo.2019.01.004.
5. Wallaert, A.; Van Loocke, W.; Hernandez, L.; Taghon, T.; Speleman, F.; Van Vlierberghe, P. Comprehensive miRNA expression profiling in human T-cell acute lymphoblastic leukemia by small RNA-sequencing. Sci. Rep. 2017, 7. https://doi.org/10.1038/S41598-017-08148-X.
6. Bhatia, S.; Kaul, D.; Varma, N. Functional genomics of tumor suppressor miR-196b in T-cell acute lymphoblastic leukemia. Mol. Cell. Biochem. 2011, 346, 103–116. https://doi.org/10.1007/s11010-010-0597-0.
7. Nemes, K.; Csóka, M.; Nagy, N.; Márk, Á.; Váradi, Z.; Dankó, T.; Kovács, G.; Kopper, L.; Sebestyén, A. Expression of Certain Leukemia/Lymphoma Related microRNAs and its Correlation with Prognosis in Childhood Acute Lymphoblastic Leukemia. Pathol. Oncol. Res. 2015, 21, 597–604. https://doi.org/10.1007/s12253-014-9861-z.
8. Li, S.W.; Li, H.; Zhang, Z.P.; Zhuo, F.; Li, Z.X. Expression and Clinical Significance of MiR-146a and MiR-221 in Childhood Acute T Lymphoblastic Leukemia. Zhongguo Shi Yan Xue Ye Xue Za Zhi 2020, 28, 436–441. https://doi.org/10.19746/j.cnki.issn.1009-2137.2020.02.013.

9. Gimenes-Teixeira, H.L.; Lucena-Araujo, A.R.; dos Santos, G.A.; Zanette, D.L.; Scheucher, P.S.; Oliveira, L.C.; Dalmazzo, L.F.; Silva-Júnior, W.A.; Falcão, R.P.; Rego, E.M. Increased expression of miR-221 is associated with shorter overall survival in T-cell acute lymphoid leukemia. Exp. Hematol. Oncol. 2013, 2. https://doi.org/10.1186/2162-3619-2-10.
10. Liu, X.; Zhang, H.; Zhang, B.; Zhang, X. Expression and role of microRNA-663b in childhood acute lymphocytic leukemia and its mechanism. Open Med. 2019, 14, 863–871. https://doi.org/10.1515/med-2019-0101.
11. Malik, D.; Kaul, D.; Chauhan, N.; Marwaha, R.K. MiR-2909-mediated regulation of KLF4: A novel molecular mechanism for differentiating between B-cell and T-cell pediatric acute lymphoblastic leukemias. Mol. Cancer 2014, 13. https://doi.org/10.1186/1476-4598-13-175.
12. Oliveira, L.H.; Schiavinato, J.L.; Fráguas, M.S.; Lucena-Araujo, A.R.; Haddad, R.; Araújo, A.G.; Dalmazzo, L.F.; Rego, E.M.; Covas, D.T.; Zago, M.A.; et al. Potential roles of microRNA-29a in the molecular pathophysiology of T-cell acute lymphoblastic leukemia. Cancer Sci. 2015, 106, 1264–1277. https://doi.org/10.1111/cas.12766.
13. Hassan, N.M.; Refaat, L.A.; Ismail, G.N.; Abdellateif, M.; Fadel, S.A.; AbdelAziz, R.S. Diagnostic, prognostic and predictive values of miR-100 and miR-210 in pediatric acute lymphoblastic Leukemia. Hematol. (United Kingdom) 2020, 25, 405–413. https://doi.org/10.1080/16078454.2020.1843753.
14. Chen, L.; Shi, Y.; Li, J.; Yang, X.; Li, R.; Zhou, X.; Zhu, L. LncRNA CDKN2B-AS1 contributes to tumorigenesis and chemoresistance in pediatric T-cell acute lymphoblastic leukemia through miR-335-3p/TRAF5 axis. Anticancer. Drugs 2020. https://doi.org/10.1097/CAD.0000000000001001.

15. Li, X.; Sanda, T.; Thomas Look, A.; Novina, C.D.; von Boehmer, H. Repression of tumor suppressor miR-451 is essential for NOTCH1-induced oncogenesis in T-ALL. J. Exp. Med. 2011, 208, 663–675. https://doi.org/10.1084/jem.20102384.
16. Luna-Aguirre, C.M.; Martinez-Fierro, M.D.L.L.; Mar-Aguilar, F.; Garza-Veloz, I.; Treviño-Alvaradod, V.; Rojas-Martinez, A.; Jaime-Perez, J.C.; Malagon-Santiago, G.I.; Gutierrez-Aguirre, C.H.; Gonzalez-Llano, O.; et al. Circulating microRNA expression profile in B-cell acute lymphoblastic leukemia. Cancer Biomarkers 2015, 15, 299–310. https://doi.org/10.3233/CBM-150465.
17. Ramani, R.; Megason, G.; Schallheim, J.; Karlson, C.; Vijayakumar, V.; Vijayakumar, S.; Hicks, C. Integrative analysis of microRNA-mediated gene signatures and pathways modulating white blood cell count in childhood acute lymphoblastic leukemia. Biomark. Insights 2017, 12. https://doi.org/10.1177/1177271917702895.
18. Rzepiel, A.; Kutszegi, N.; Gézsi, A.; Sági, J.C.; Egyed, B.; Péter, G.; Butz, H.; Nyíro, G.; Müller, J.; Kovács, G.T.; et al. Circulating microRNAs as minimal residual disease biomarkers in childhood acute lymphoblastic leukemia. J. Transl. Med. 2019, 17. https://doi.org/10.1186/s12967-019-2114-x.
19. Labib, H.A.; Elantouny, N.G.; Ibrahim, N.F.; Alnagar, A.A. Upregulation of microRNA-21 is a poor prognostic marker in patients with childhood B cell acute lymphoblastic leukemia. Hematology 2017, 22, 392–397. https://doi.org/10.1080/10245332.2017.1292204.
20. Xue, Y.; Yang, X.; Hu, S.; Kang, M.; Chen, J.; Fang, Y. A genetic variant in miR-100 is a protective factor of childhood acute lymphoblastic leukemia. Cancer Med. 2019, 8, 2553–2560. https://doi.org/10.1002/cam4.2082.
21. Ju, X.; Li, D.; Shi, Q.; Hou, H.; Sun, N.; Shen, B. Differential microRNA expression in childhood B-cell precursor acute lymphoblastic leukemia. Pediatr. Hematol. Oncol. 2009, 26, 1–10. https://doi.org/10.1080/08880010802378338.

22. Li, X.; Li, D.; Zhuang, Y.; Shi, Q.; Wei, W.; Zhang, H.; Ju, X. li [The expression and regulatory mechanism of microRNA-708 in pediatric common B-cell acute lymphoblastic leukemia]. Zhonghua Xue Ye Xue Za Zhi 2013, 34, 138–143.
23. Lou, Y.; Liu, L.; Zhan, L.; Wang, X.; Fan, H. MIR-187-5p regulates cell growth and apoptosis in acute lymphoblastic Leukemia via DKK2. Oncol. Res. 2016, 24, 89–97. https://doi.org/10.3727/096504016X14597766487753.
24. de Oliveira, J.C.; Scrideli, C.A.; Brassesco, M.S.; Yunes, J.A.; Brandalise, S.R.; Tone, L.G. MiR-708-5p is differentially expressed in childhood acute lymphoblastic leukemia but not strongly associated to clinical features. Pediatr. Blood Cancer 2015, 62, 177–178. https://doi.org/10.1002/pbc.25222.
25. Schotte, D.; Chau, J.C.K.; Sylvester, G.; Liu, G.; Chen, C.; van der Velden, V.H.J.; Broekhuis, M.J.C.; Peters, T.C.J.M.; Pieters, R.; den Boer, M.L. Identification of new microRNA genes and aberrant microRNA profiles in childhood acute lymphoblastic leukemia. Leukemia 2009, 23, 313–322. https://doi.org/10.1038/leu.2008.286.
26. Sheybani, Z.; Rahgozar, S.; Ghodousi, E.S. The hedgehog signal transducer smoothened and microRNA-326: Pathogenesis and regulation of drug resistance in pediatric B-cell acute lymphoblastic leukemia. Cancer Manag. Res. 2019, 11, 7621–7630. https://doi.org/10.2147/CMAR.S214405.
27. Tian, L.; Cao, J.; Ji, Q.; Zhang, C.; Qian, T.; Song, X.; Huang, B.; Tian, X. The downregulation of miR-3173 in B-cell acute lymphoblastic leukaemia promotes cell invasion via PTK2. Biochem. Biophys. Res. Commun. 2017, 494, 569–574. https://doi.org/10.1016/j.bbrc.2017.10.013.

28. Zhu, Y.-Y.; Wu, R.-N.; Li, X.; Chen, X.-B. Value of serum miR-922 and miR-506 expression levels in the diagnosis and prognostic assessment of childhood acute lymphoblastic leukemia. Zhongguo Dang Dai Er Ke Za Zhi 2021, 23, 1021–1026. https://doi.org/10.7499/J.ISSN.1008-8830.2105148.
29. Wu, Z.; Eguchi-Ishimae, M.; Yagi, C.; Iwabuki, H.; Gao, W.; Tauchi, H.; Inukai, T.; Sugita, K.; Ishii, E.; Eguchi, M. HMGA2 as a potential molecular target in KMT2A-AFF1-positive infant acute lymphoblastic leukaemia. Br. J. Haematol. 2015, 171, 818–829. https://doi.org/10.1111/bjh.13763.
30. Nishi, M.; Eguchi-Ishimae, M.; Wu, Z.; Gao, W.; Iwabuki, H.; Kawakami, S.; Tauchi, H.; Inukai, T.; Sugita, K.; Hamasaki, Y.; et al. Suppression of the let-7b microRNA pathway by DNA hypermethylation in infant acute lymphoblastic leukemia with MLL gene rearrangements. Leukemia 2013, 27, 389–397. https://doi.org/10.1038/leu.2012.242.
31. Stumpel, D.J.P.M.; Schotte, D.; Lange-Turenhout, E.A.M.; Schneider, P.; Seslija, L.; De Menezes, R.X.; Marquez, V.E.; Pieters, R.; Den Boer, M.L.; Stam, R.W. Hypermethylation of specific microRNA genes in MLL-rearranged infant acute lymphoblastic leukemia: Major matters at a micro scale. Leukemia 2011, 25, 429–439. https://doi.org/10.1038/leu.2010.282.
32. Li, X.J.; Luo, X.Q.; Han, B.W.; Duan, F.T.; Wei, P.P.; Chen, Y.Q. MicroRNA-100/99a, deregulated in acute lymphoblastic leukaemia, suppress proliferation and promote apoptosis by regulating the FKBP51 and IGF1R/mTOR signalling pathways. Br. J. Cancer 2013, 109, 2189–2198. https://doi.org/10.1038/bjc.2013.562.

33. Akbari Moqadam, F.; Boer, J.M.; Lange-Turenhout, E.A.M.; Pieters, R.; Den Boer, M.L. Altered expression of miR-24, miR-126 and miR-365 does not affect viability of childhood TCF3-rearranged leukemia cells. Leukemia 2014, 28, 1008–1014. https://doi.org/10.1038/leu.2013.308.
34. Almeida, R.S.; Costa e Silva, M.; Coutinho, L.L.; Garcia Gomes, R.; Pedrosa, F.; Massaro, J.D.; Donadi, E.A.; Lucena-Silva, N. MicroRNA expression profiles discriminate childhood T- from B-acute lymphoblastic leukemia. Hematol. Oncol. 2019, 37, 103–112. https://doi.org/10.1002/hon.2567.
35. Swellam, M.; El-Khazragy, N. Clinical impact of circulating microRNAs as blood-based marker in childhood acute lymphoblastic leukemia. Tumor Biol. 2016, 37, 10571–10576. https://doi.org/10.1007/s13277-016-4948-7.
36. Schotte, D.; de Menezes, R.X.; Moqadam, F.A.; Khankahdani, L.M.; Lange-Turenhout, E.; Chen, C.; Pieters, R.; den Boer, M.L. MicroRNA characterize genetic diversity and drug resistance in pediatric acute lymphoblastic leukemia. Haematologica 2011, 96, 703–711. https://doi.org/10.3324/haematol.2010.026138.
37. Avigad, S.; Verly, I.R.; Lebel, A.; Kordi, O.; Shichrur, K.; Ohali, A.; Hameiri-Grossman, M.; Kaspers, G.J.; Cloos, J.; Fronkova, E.; et al. miR expression profiling at diagnosis predicts relapse in pediatric precursor B-cell acute lymphoblastic leukemia. Genes Chromosom. Cancer 2016, 55, 328–339. https://doi.org/10.1002/gcc.22334.
38. De Oliveira, J.C.; Scrideli, C.A.; Brassesco, M.S.; Morales, A.G.; Pezuk, J.A.; Queiroz, R. de P.; Yunes, J.A.; Brandalise, S.R.; Tone, L.G. Differential MiRNA expression in childhood acute lymphoblastic leukemia and association with clinical and biological features. Leuk. Res. 2012, 36, 293–298. https://doi.org/10.1016/j.leukres.2011.10.005.

39. Schotte, D.; Lange-Turenhout, E.A.M.; Stumpel, D.J.P.M.; Stam, R.W.; Buijs-Gladdines, J.G.C.A.M.; Meijerink, J.P.P.; Pieters, R.; den Boer, M.L. Expression of miR-196b is not exclusively MLL-driven but is especially linked to activation of HOXA genes in pediatric acute lymphoblastic leukemia. Haematologica 2010, 95, 1675–1682. https://doi.org/10.3324/haematol.2010.023481.
40. Krzanowski, J.; Madzio, J.; Pastorczak, A.; Tracz, A.; Braun, M.; Tabarkiewicz, J.; Pluta, A.; Młynarski, W.; Zawlik, I. Selected miRNA levels are associated with IKZF1 microdeletions in pediatric acute lymphoblastic leukemia. Oncol. Lett. 2017, 14, 3853–3861. https://doi.org/10.3892/ol.2017.6599.
41. Schotte, D.; Moqadam, F.A.; Lange-Turenhout, E.A.M.; Chen, C.; Van Ijcken, W.F.J.; Pieters, R.; Den Boer, M.L. Discovery of new microRNAs by small RNAome deep sequencing in childhood acute lymphoblastic leukemia. Leukemia 2011, 25, 1389–1399. https://doi.org/10.1038/leu.2011.105.
42. El-maadawy, E.A.; Bakry, R.M.; Moussa, M.M.; El-Naby, S.H.; Talaat, R.M. Alteration in miRNAs expression in paediatric acute lymphocyticleukaemia: Insight into patients' therapeutic response. Clin. Exp. Pharmacol. Physiol. 2021, 48, 35–43. https://doi.org/10.1111/1440-1681.13386.
43. Piatopoulou, D.; Avgeris, M.; Marmarinos, A.; Xagorari, M.; Baka, M.; Doganis, D.; Kossiva, L.; Scorilas, A.; Gourgiotis, D. MiR-125b predicts childhood acute lymphoblastic leukaemia poor response to BFM chemotherapy treatment. Br. J. Cancer 2017, 117, 801–812. https://doi.org/10.1038/bjc.2017.256.

44. Han, B.W.; Feng, D.D.; Li, Z.G.; Luo, X.Q.; Zhang, H.; Li, X.J.; Zhang, X.J.; Zheng, L.L.; Zeng, C.W.; Lin, K.Y.; et al. A set of miRNAs that involve in the pathways of drug resistance and leukemic stem-cell differentiation is associated with the risk of relapse and glucocorticoid response in childhood ALL. Hum. Mol. Genet. 2011, 20, 4903–4915. https://doi.org/10.1093/hmg/ddr428.
45. Akbari Moqadam, F.; Lange-Turenhout, E.A.M.; Van Der Veer, A.; Marchante, J.R.M.; Boer, J.M.; Pieters, R.; Den Boer, M. MicroRNA signature in BCR-ABL1-like and BCR-ABL1-positive childhood acute lymphoblastic leukemia: Similarities and dissimilarities. Leuk. Lymphoma 2014, 55, 1942–1945. https://doi.org/10.3109/10428194.2013.858813.
46. Gefen, N.; Binder, V.; Zaliova, M.; Linka, Y.; Morrow, M.; Novosel, A.; Edry, L.; Hertzberg, L.; Shomron, N.; Williams, O.; et al. Hsa-mir-125b-2 is highly expressed in childhood ETV6/RUNX1 (TEL/AML1) leukemias and confers survival advantage to growth inhibitory signals independent of p53. Leukemia 2010, 24, 89–96. https://doi.org/10.1038/leu.2009.208.
47. Busche, S.; Ge, B.; Vidal, R.; Spinella, J.F.; Saillour, V.; Richer, C.; Healy, J.; Chen, S.H.; Droit, A.; Sinnett, D.; et al. Integration of high-resolution methylome and transcriptome analyses to dissect epigenomic changes in childhood acute lymphoblastic leukemia. Cancer Res. 2013, 73, 4323–4336. https://doi.org/10.1158/0008-5472.CAN-12-4367.
48. Yang, Y.L.; Yen, C.T.; Pai, C.H.; Chen, H.Y.; Yu, S.L.; Lin, C.Y.; Hu, C.Y.; Jou, S.T.; Lin, D.T.; Lin, S.R.; et al. A double negative loop comprising ETV6/RUNX1 and MIR181A1 contributes to differentiation block in t(12;21)-positive acute lymphoblastic leukemia. PLoS ONE 2015, 10. https://doi.org/10.1371/journal.pone.0142863.

49. Diakos, C.; Zhong, S.; Xiao, Y.; Zhou, M.; Vasconcelos, G.M.; Krapf, G.; Yeh, R.F.; Zheng, S.; Kang, M.; Wiencke, J.K.; et al. TEL-AML1 regulation of survivin and apoptosis via miRNA-494 and miRNA-320a. Blood 2010, 116, 4885–4893. https://doi.org/10.1182/blood-2009-02-206706.
50. Yan, J.; Jiang, N.; Huang, G.; Tay, J.L.S.; Lin, B.; Bi, C.; Koh, G.S.; Li, Z.; Tan, J.; Chung, T.H.; et al. Deregulated MIR335 that targets MAPK1 is implicated in poor outcome of paediatric acute lymphoblastic leukaemia. Br. J. Haematol. 2013, 163, 93–103. https://doi.org/10.1111/bjh.12489.
51. Scherr, M.; Elder, A.; Battmer, K.; Barzan, D.; Bomken, S.; Ricke-Hoch, M.; Schröder, A.; Venturini, L.; Blair, H.J.; Vormoor, J.; et al. Differential expression of miR-17~92 identifies BCL2 as a therapeutic target in BCR-ABL-positive B-lineage acute lymphoblastic leukemia. Leukemia 2014, 28, 554–565. https://doi.org/10.1038/leu.2013.361.
52. Shafik, R.E.; Abd El Wahab, N.; Senoun, S.A.; Ebeid, E.; El Taweel, M.A. Expression of micro-RNA 128 and let-7b in pediatric acute lymphoblastic leukemia cases. Asian Pacific J. Cancer Prev. 2018, 19, 2263–2267. https://doi.org/10.22034/APJCP.2018.19.8.2263.
53. Vendramini, E.; Giordan, M.; Giarin, E.; Michielotto, B.; Fazio, G.; Cazzaniga, G.; Biondi, A.; Silvestri, D.; Valsecchi, M.G.; Muckenthaler, M.U.; et al. High expression of miR-125b-2 and SNORD116 noncoding RNA clusters characterize ERG-related B cell precursor acute lymphoblastic leukemia. Oncotarget 2017, 8, 42398–42413. https://doi.org/10.18632/oncotarget.16392.
54. Renou, L.; Boelle, P.Y.; Deswarte, C.; Spicuglia, S.; Benyoucef, A.; Calvo, J.; Uzan, B.; Belhocine, M.; Cieslak, A.; Landman-Parker, J.; et al. Homeobox protein TLX3 activates miR-125b expression to promote T-cell acute lymphoblastic leukemia. Blood Adv. 2017, 1, 733–747. https://doi.org/10.1182/bloodadvances.2017005538.

55. Wang, L.; Zhang, H.; Lei, D. Microrna-146a promotes growth of acute leukemia cells by downregulating ciliary neurotrophic factor receptor and activating jak2/stat3 signaling. Yonsei Med. J. 2019, 60, 924–934. https://doi.org/10.3349/ymj.2019.60.10.924.
56. Agueli, C.; Cammarata, G.; Salemi, D.; Dagnino, L.; Nicoletti, R.; La Rosa, M.; Messana, F.; Marfia, A.; Bica, M.G.; Coniglio, M.L.; et al. 14q32/miRNA clusters loss of heterozygosity in acute lymphoblastic leukemia is associated with up-regulation of BCL11a. Am. J. Hematol. 2010, 85, 575–578. https://doi.org/10.1002/ajh.21758.
57. Amankwah, E.K.; Devidas, M.; Teachey, D.T.; Rabin, K.R.; Brown, P.A. Six Candidate miRNAs Associated With Early Relapse in Pediatric B-Cell Acute Lymphoblastic Leukemia. Anticancer Res. 2020, 40, 3147–3153. https://doi.org/10.21873/anticanres.14296.
58. Gutierrez-Camino, A.; Richer, C.; St-Onge, P.; Lopez-Lopez, E.; Bañeres, A.C.; De Andoin, N.G.; Sastre, A.; Astigarraga, I.; Martin-Guerrero, I.; Sinnett, D.; et al. Role of rs10406069 in miR-5196 in hyperdiploid childhood acute lymphoblastic leukemia. Epigenomics 2020, 12, 1949–1955. https://doi.org/10.2217/epi-2020-0152.
59. Alsuwaidi, L.; Hachim, M.; Senok, A. Novel Markers in Pediatric Acute Lymphoid Leukemia: The Role of ADAM6 in B Cell Leukemia. Front. Cell Dev. Biol. 2021, 9. https://doi.org/10.3389/fcell.2021.706129.
60. Kotani, A.; Ha, D.; Hsieh, J.; Rao, P.K.; Schotte, D.; Den Boer, M.L.; Armstrong, S.A.; Lodish, H.F. miR-128b is a potent glucocorticoid sensitizer in MLL-AF4 acute lymphocytic leukemia cells and exerts cooperative effects with miR-221. Blood 2009, 114, 4169–4178. https://doi.org/10.1182/blood-2008-12-191619.

61. Doerrenberg, M.; Kloetgen, A.; Hezaveh, K.; Wössmann, W.; Bleckmann, K.; Stanulla, M.; Schrappe, M.; McHardy, A.C.; Borkhardt, A.; Hoell, J.I. T-cell acute lymphoblastic leukemia in infants has distinct genetic and epigenetic features compared to childhood cases. Genes Chromosom. Cancer 2017, 56, 159–167. https://doi.org/10.1002/gcc.22423.
62. Piroozian, F.; Bagheri Varkiyani, H.; Koolivand, M.; Ansari, M.; Afsa, M.; AtashAbParvar, A.; MalekZadeh, K. The impact of variations in transcription of DICER and AGO2 on exacerbation of childhood B-cell lineage acute lymphoblastic leukaemia. Int. J. Exp. Pathol. 2019, 100, 184–191. https://doi.org/10.1111/iep.12316.
